# Visualizing Conformational Space of Functional Biomolecular Complexes by Deep Manifold Learning

**DOI:** 10.3390/ijms23168872

**Published:** 2022-08-09

**Authors:** Zhaolong Wu, Enbo Chen, Shuwen Zhang, Yinping Ma, Youdong Mao

**Affiliations:** 1 State Key Laboratory for Artificial Microstructure and Mesoscopic Physics, School of Physics, Peking University, Beijing 100871, China; 2 Peking-Tsinghua Joint Center for Life Sciences, Academy of Advanced Interdisciplinary Studies, Peking University, Beijing 100871, China; 3 Center for Quantitative Biology, Academy of Advanced Interdisciplinary Studies, Peking University, Beijing 100871, China; 4 Computing Center, Peking University, Beijing 100871, China; 5 National Biomedical Imaging Center, Peking University, Beijing 100871, China

**Keywords:** AlphaCryo4D, cryogenic electron microscopy, biomolecular complex, structural dynamics, conformational space, energy landscape, deep learning, manifold learning, particle voting

## Abstract

The cellular functions are executed by biological macromolecular complexes in nonequilibrium dynamic processes, which exhibit a vast diversity of conformational states. Solving the conformational continuum of important biomolecular complexes at the atomic level is essential to understanding their functional mechanisms and guiding structure-based drug discovery. Here, we introduce a deep manifold learning framework, named AlphaCryo4D, which enables atomic-level cryogenic electron microscopy (cryo-EM) reconstructions that approximately visualize the conformational space of biomolecular complexes of interest. AlphaCryo4D integrates 3D deep residual learning with manifold embedding of pseudo-energy landscapes, which simultaneously improves 3D classification accuracy and reconstruction resolution via an energy-based particle-voting algorithm. In blind assessments using simulated heterogeneous datasets, AlphaCryo4D achieved 3D classification accuracy three times those of alternative methods and reconstructed continuous conformational changes of a 130-kDa protein at sub-3 Å resolution. By applying this approach to analyze several experimental datasets of the proteasome, ribosome and spliceosome, we demonstrate its potential generality in exploring hidden conformational space or transient states of macromolecular complexes that remain hitherto invisible. Integration of this approach with time-resolved cryo-EM further allows visualization of conformational continuum in a nonequilibrium regime at the atomic level, thus potentially enabling therapeutic discovery against highly dynamic biomolecular targets.

## 1. Introduction

Essential cellular functions are executed and regulated by macromolecular complexes comprising many subunits, such as the ribosome, proteasome, replisome, and spliceosome. Their structures in functional cycles are often compositionally heterogeneous and conformationally dynamic, involving many reversibly associated components in cells. Visualizing the conformational continuum of highly dynamic megadalton complexes at high resolution is crucial for understanding their functional mechanisms and for guiding structure-based therapeutic development. The current approaches in cryogenic electron microscopy (cryo-EM) structure determination allow atomic-level visualization of major conformational states of these dynamic complexes [1,2,3,4,5,6,7,8,9,10,11,12]. However, high-resolution structure determination of transient, nonequilibrium intermediates connecting their major states during their functional cycles has been prohibitory at large. To date, there has been a lack of appropriate approaches to visualizing the conformational space of large biomolecular complexes at high resolution.

The problem of structural heterogeneity in cryo-EM structure determination has been investigated with numerous computational approaches including maximum-likelihood-based classification and multivariate statistical analysis [5,6,11,13,14,15,16,17,18]. As an extension of multivariate statistical analysis, several machine-learning approaches were recently proposed to estimate a continuous conformational distribution in the latent space [11,12,19,20,21,22]. The estimation of continuous conformational distributions does not guarantee an improvement of 3D classification accuracy nor warrant the identification of hidden conformational states of biological importance. These methods often trade off the reconstruction resolution for gaining an overall representation of the conformational landscape in the latent space. The insufficient resolution would preclude their potential applications in structure-based drug discovery.

The most widely used method so far to counteract structural heterogeneity in cryo-EM for resolution improvement is the hierarchical maximum-likelihood-based 3D classification due to its ease of application [1,23,24]. To improve the resolution of major conformers, low-quality classes are often manually removed during iterations of curated hierarchical classification, inevitably causing an incomplete representation of the true conformational landscape [25,26]. The outcome of refined cryo-EM maps depends on user expertise in decision-making in class selection [27], which can be biased by user experience and subjectivity. Importantly, a considerable portion of misclassified images can limit the achievable resolution of observed conformational states and result in missing states that could be biologically important.

In addition to the approaches characterizing global structural heterogeneity, other methods were proposed to analyze local structural dynamics or to improve the local resolution of flexible regions in cryo-EM reconstructions. These include the multi-body or focused refinement [4,28,29], normal mode analysis [30,31], flexible refinement [32,33], non-uniform refinement [34] and integrating traditional molecular dynamics (MD) simulations with or without use of deep learning [35,36]. Nonetheless, these methods were not designed to recover a complete picture of structural dynamics hidden in cryo-EM datasets.

The energy landscape is a statistical physical representation of the conformational space of a macromolecular complex and is the basis of the transition-state theory of chemical reaction dynamics [37,38]. The minimum-energy path (MEP) on the energy landscape theoretically represents the most probable trajectory of conformational transitions and can inform the activation energy for chemical reactions [38,39]. Previous studies have demonstrated the benefit of pseudo-energy landscape estimation in characterizing conformational variation of macromolecules from cryo-EM data [40,41,42,43,44,45,46]. In these studies, single-particle images classified into the same conformation were assumed to have approximately similar free energy. Thus, the relative energy differences between distinct conformations could be computed by the ratio of particle numbers classified into different conformers using the Boltzmann relation, provided that the molecular system can be approximately described as a canonical ensemble. Because of inaccuracy in 3D classification, a potential violation of canonical ensemble approximation and possible compositional heterogeneity, such a procedure may be at best considered to yield a pseudo-energy landscape.

The combination of linear dimensionality reduction via 3D principal component analysis (PCA) and pseudo-energy landscape estimation has been demonstrated to be capable of capturing the overall conformational landscape [41,42,43]. The benefit of using nonlinear dimensionality reduction and manifold embedding, such as diffusion map [47], to replace PCA for estimating pseudo-energy landscapes has also been investigated at a limited resolution, with the assumption that the conformational changes can be discerned through a narrow angular aperture [40,44,45]. This assumption restricts its potential applications to more complicated conformational dynamics.

Breaking the resolution barrier in visualizing the conformational space of highly dynamic complexes represents a formidable technical challenge. Achieving this goal may further leverage cryo-EM applications in both basic biomedical science and drug discovery, where high-resolution features of highly dynamic components may provide crucial mechanistic insights and guide more accurate structure-based ligand design. This would require a substantial improvement in 3D classification techniques that are optimized for achieving higher resolution. We hypothesize that a high-quality estimation of the pseudo-energy landscape could be potentially used to improve the 3D classification of highly heterogeneous cryo-EM datasets. If different conformers or continuous conformational changes can be sufficiently mapped and differentiated on a pseudo-energy landscape, the energetic visualization of the conformational continuum could then be used to discover previously ‘invisible’ conformers and achieve higher resolution for lowly populated, transient, or nonequilibrium states. 

To explore these ideas in this study, we developed a novel machine learning framework named AlphaCryo4D that can break the existing limitations and enable 4D cryo-EM reconstruction of highly dynamic, lowly populated intermediates or transient states at the atomic level. We examined the general applicability of AlphaCryo4D in analyzing several large synthetic datasets over a wide range of signal-to-noise ratios (SNRs) and four experimental cryo-EM datasets of diverse sample behaviors in complex dynamics, including the human 26S proteasome [25], malaria parasite 80S ribosome [48], pre-catalytic spliceosome [49] and bacterial ribosomal assembly intermediates [50]. Our approach pushes the envelope of visualizing the conformational space of macromolecular complexes beyond the previously achieved scope toward the atomic level.

## 2. Results

### 2.1. Design of Deep Manifold Learning

**Figure 1:**
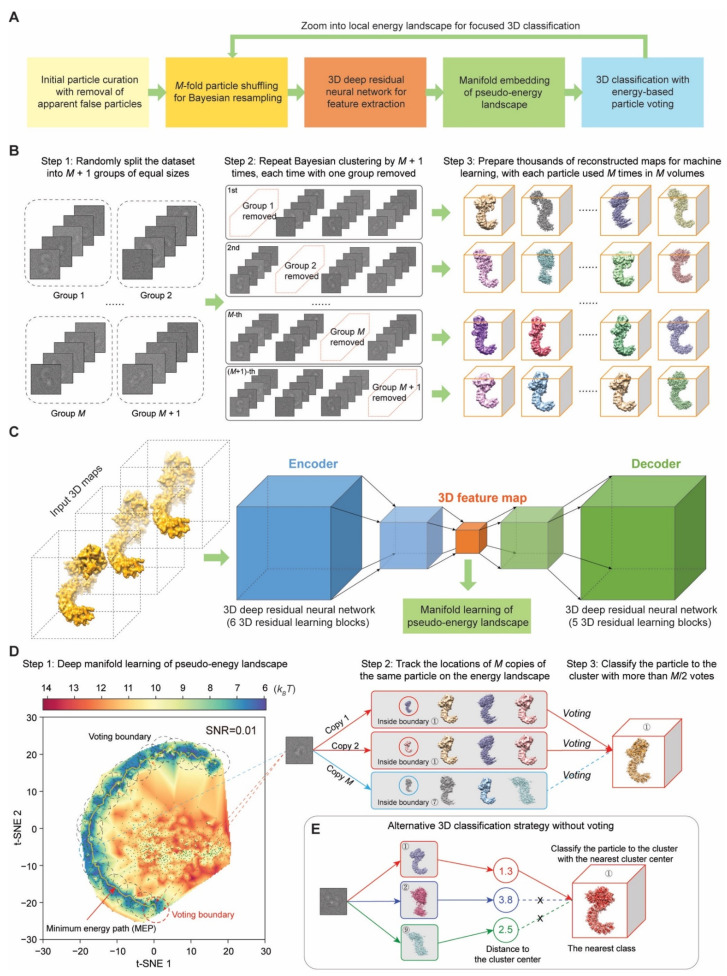
Algorithmic framework of AlphaCryo4D for 4D cryo-EM reconstruction. (**A**) Schematic showing the major conceptual steps of single-particle cryo-EM data processing in AlphaCryo4D. (**B**) Schematic showing the method of *M*-fold particle shuffling for resampling of 3D volumes. In step 1, all particles are split randomly into *M* + 1 groups equally. Then step 2 carries out the Bayesian clustering for reconstructions of 3D density maps within each of the *M* + 1 particle sets that are shuffled via the ‘removing-one-group’ approach. After these two steps, thousands of 3D volumes are generated for the subsequent 3D deep learning, with each particle contributing to *M* volumes. (**C**) Schematic of unsupervised deep residual learning of 3D feature maps by a six-block autoencoder conjugated to a five-block decoder. (**D**) Schematic showing the algorithmic concept of energy-based particle voting for 3D classification of improved accuracy. The left panel shows the pseudo-energy landscape obtained by deep manifold learning. After clustering along the minimum energy path, all *M* locations of each particle on the pseudo-energy landscape can be tracked to cast *M* votes. A vote of the particle is only counted for the cluster when it is mapped within the voting boundary of the cluster, as indicated by the circles marked on the pseudo-energy landscape. Eventually, this particle is classified in the 3D cluster with at least *M*/2 votes for this particle. (**E**) Alternative distance-based 3D classification method. This serves as a control in the analysis of the algorithmic performance. Instead of particle voting, each particle is directly classified to the cluster with the nearest clustering center among the *M* volume data points in the distance-based 3D classification strategy.

The conceptual framework of AlphaCryo4D integrates unsupervised deep learning with manifold embedding to learn a pseudo-energy landscape, which directs cryo-EM reconstructions of the conformational continuum or transient states via an energy-based particle-voting algorithm. AlphaCryo4D consists of four major steps (Figure 1A). First, hundreds to thousands of 3D volumes are resampled with *M*-fold particle shuffling and Bayesian clustering using a maximum-likelihood-based 3D (ML3D) classification algorithm implemented in RELION [3,7]. To this end, all curated particles are aligned in a common frame of reference through a consensus 3D refinement. They are then randomly divided into *M* + 1 groups of equal data sizes. In the step of ‘*M*-fold particle shuffling’, one group of particles is excluded to form a shuffled dataset. This procedure is repeated *M* + 1 times, each time with a different particle group being excluded, resulting in *M* + 1 shuffled datasets (Figure 1B). Each shuffled dataset is subject to 3D volume resampling separately and is clustered into tens to hundreds of 3D reconstructions through Bayesian clustering in RELION [3,7]. In total, hundreds to thousands of volumes from all shuffled datasets are expected to be resampled through these steps.

**Figure 2:**
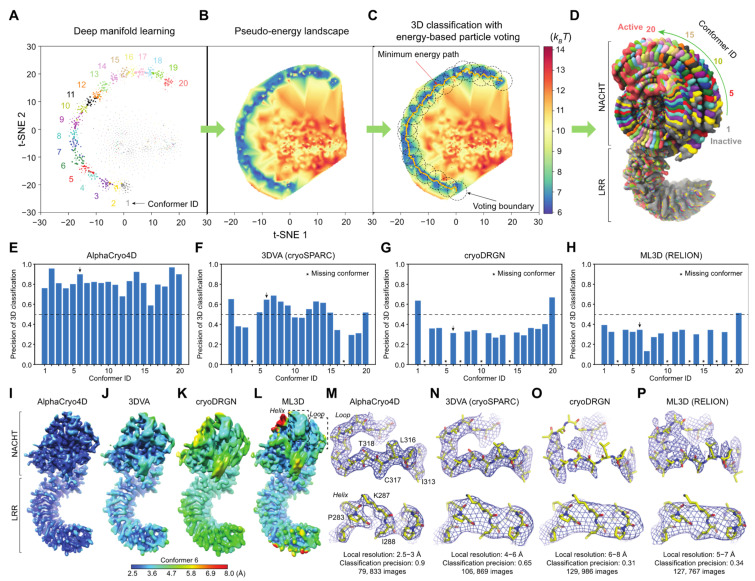
Performance evaluation of AlphaCryo4D for reconstruction of the conformational continuum at the atomic level. (**A**) Manifold learning of 3D volumes of the simulated NLRP3 dataset with SNR of 0.01 by the 3D autoencoder. Each data point corresponds to a 3D volume. The color labels the conformer identity of ground truth for the purpose of verification. (**B**) Pseudo-energy landscape computed from the manifold embedding shown in (**A**). (**C**) Minimum energy path (orange line) calculated by the string method is used to find the approximate cluster centers of 20 conformers. The cluster boundaries for energy-based particle voting are shown as dashed circles. (**D**) The 20 reconstructions resulting from AlphaCryo4D using a simulated dataset with SNR of 0.01 are superimposed together, shown in surface representation, and aligned against its LRR domain. (**E**–**H**) Plots of 3D classification precisions of the 20 NLRP3 conformers from blind assessments on the simulated NLRP3 dataset with SNR of 0.01, using AlphaCyo4D (**E**), 3DVA in cryoSPARC (**F**) [11], cryoDRGN (**G**) [12], and ML3D in RELION (**H**) [3]. All 3D conformers in panel (**E**) were reconstructed to 2.6–2.9 Å resolution (Supplementary Figure S2D). Asterisks mark the missing conformers that were completely lost due to misclassification. Black arrows highlight Conformer 6. (**I**–**L**) Typical side-by-side comparison of density map quality and local resolution of the same conformer (ID 6) reconstructed by AlphaCryo4D (**I**), 3DVA (**J**), cryoDRGN (**K**) and ML3D (**L**). The maps are colored by their local resolutions calculated by Bsoft blocres program. (**M**–**P**) Closeup side-by-side comparison of the same two secondary structures, including a loop (upper row) and a helix (lower row), in the NACHT domain illustrates considerable improvements in local density quality and resolution by AlphaCryo4D (**M**) as opposed to 3DVA (**N**), cryoDRGN (**O**) and ML3D (**P**). The locations of the loop and helix in the NLRP3 structure are marked by dashed boxes in panel (**L**). The same ground-truth atomic model of Conformer 6 shown in stick representations is superimposed with the density maps shown in blue mesh representations, all from the same perspective. The atomic model was not further refined against each map for visual validation of the map accuracy.

Next, all resampled volumes are learned by a 3D autoencoder made of a deep residual convolutional neural network in an unsupervised fashion (Figure 1C) [51,52]. The 3D deep residual autoencoder is composed of six and five residual learning blocks in the encoder and decoder, respectively. Each residual learning block is derived from the ResNet architecture and consists of two convolutional neural network layers with shortcut connection skipping two adjacent layers, which has been shown to mitigate the degradation problem that learning accuracy becomes saturated and then quickly degrades with increasing the network depth [51,52]. A 3D feature map is extracted for each volume by the deep residual autoencoder and is concatenated with the volume data for nonlinear dimensionality reduction by manifold embedding with the t-distributed stochastic neighbor embedding (t-SNE) algorithm (Figure 1D and Figure 2A,B) [53].

Third, the relative free-energy difference between 3D volumes is estimated using the particle number of each volume via the Boltzmann relation [40]. A pseudo-energy landscape is visualized by mapping the values of energy difference on the learned manifold [40,41,42,43,44,45,46]. Note, that only the relative difference of free energy can be estimated, and the estimated pseudo-energy values do not represent the total energy of the system, which is not measurable by the Boltzmann relation. Then, a string method can be optionally used to search the MEP on the pseudo-energy landscape [54,55]. The local energy minima or transition states connecting adjacent minimum-energy states can be defined as the centers of 3D clustering, with a circular range defined as the cluster boundary for subsequent particle voting (Figure 1D and Figure 2C); 3D volumes within a cluster boundary on the pseudo-energy landscape form a 3D cluster.

Last, because each particle is used *M* times during volume resampling, it is mapped to *M* locations on the pseudo-energy landscape. The mapping of each copy of the particle is called a ‘vote’. By counting the number of votes of the same particle cast within the same cluster boundary on the pseudo-energy landscape, the reproducibility of the machine learning procedure can be evaluated at the single-particle level. Each particle is classified into the 3D cluster that receives more than *M*/2 votes of this particle within its voting boundary (Figure 1D and Figure 2C,D). If none of the 3D clusters on the pseudo-energy landscape receives more than *M*/2 votes of a given particle, the corresponding particle is voted out and excluded for further 3D reconstruction. The resulting 3D classes are expected to be conformationally homogeneous enough for high-resolution cryo-EM refinement.

Since many protein complexes exhibit profound conformational changes in different local regions, we also implemented a focused classification strategy of AlphaCryo4D that applies a local 3D mask [6] throughout the entire procedure, which is executed as an iterative step after initial 3D classification by AlphaCryo4D in the absence of any 3D mask (Figure 1A).

### 2.2. Solving Conformational Continuum at Atomic Level

To assess the numerical performance of AlphaCryo4D, we generated three large synthetic heterogeneous cryo-EM datasets with signal-to-noise ratios (SNRs) of 0.05, 0.01 and 0.005. Each dataset includes 2 million randomly oriented single particles computationally simulated from 20 hypothetical conformer models of the ~130-kDa NLRP3 inflammasome protein (PDB ID: 6NPY) [56]. These conformers imitate the conformational continuum of the NACHT domain rotating around the LRR domain over an angular range of 90° during inflammasome activation (Figure 2D) [56]. The particles for each conformer are uniformly distributed in each dataset. 

**Figure 3:**
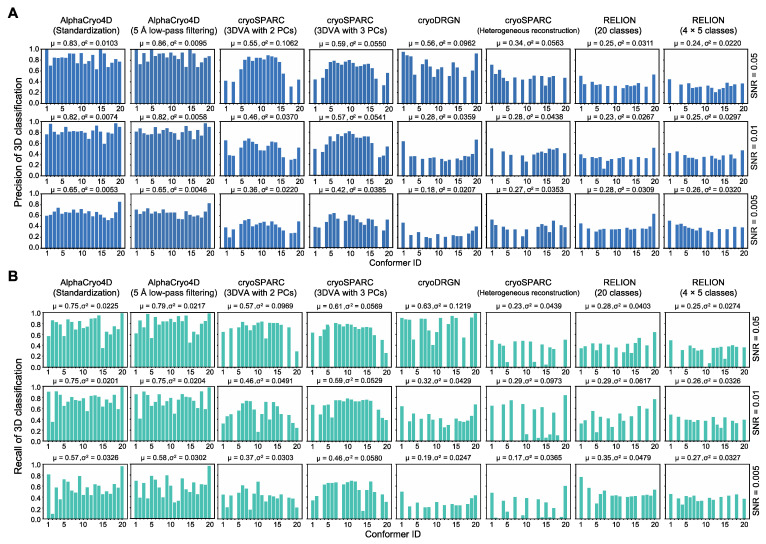
Performance comparison of AlphaCryo4D with alternative 3D classification methods using the simulated heterogeneous NLRP3 datasets of different SNRs. (**A**) 3D classification precision of the simulated datasets by AlphaCryo4D (first column), AlphaCryo4D preprocessed by 5 Å low-pass filtering instead of standardization (second column), 3DVA with two and three principal components (PCs) in cryoSPARC (third and fourth columns), cryoDRGN (fifth column), heterogeneous reconstruction in cryoSPARC (sixth column) and maximum-likelihood 3D classification in RELION (seventh column for directly classifying into 20 classes and eighth column for hierarchically classifying into four classes, each further sub-classified into five classes). (**B**) 3D classification recall of AlphaCryo4D in comparison to other methods. The recall of each state was calculated as the ratio of true positive particles in all ground truth particles. In all methods, the class number was set to 20. The results of SNRs of 0.05 (the first row), 0.01 (the second row) and 0.005 (the third row) are shown on three rows for side-by-side comparison. On the top of each panel, the symbols of μ and $\sigma^{2}$ denote the mean and variance of precision, respectively, with the values of missing classes treated as zeros. In the maximum-likelihood classification of RELION, both direct and hierarchical strategies are compared in the study.

We conducted blind assessments on 3D classification and heterogeneous reconstructions of the simulated NLRP3 datasets by AlphaCryo4D, without providing any information on particle orientations, translations, and conformational identities (Figure 2A–D and Supplementary Figure S1). The 3D classification precision of a retrieved conformer was computed as the ratio of the particle number of a correct class assignment (based on the ground truth) versus the total particle number in the class. The results were then compared with several alternative methods, including conventional maximum-likelihood-based 3D (ML3D) classification in RELION [3,4,6], 3D variability analysis (3DVA) in cryoSPARC [2,11], and deep generative model-based cryoDRGN [12]. In all blind tests by presetting the class number to 20, AlphaCryo4D retrieved all 20 conformers and markedly outperformed the alternative methods, with an average of 3D classification precision at 0.83, 0.82 and 0.65 for datasets with SNRs of 0.05, 0.01 and 0.005, respectively (Figure 2E and Figure 3). By contrast, all alternative methods missed two to seven conformers entirely and exhibited 3D classification precisions in the range of 0.2–0.5 in general (Figure 2F–H and Figure 3A and Supplementary Figures S2 and S3).

By increasing the preset class number to 25 or 30 which is more than the number of ground-truth conformers, all tested methods appear to be improved in the precision of 3D classification marginally but also reduced in the recall of classification (defined as the percentage of correctly assigned particles versus the number of ground-truth particles for a conformer) (Supplementary Figure S4). In these cases, AlphaCryo4D still outperformed all alternative methods considerably, with the highest average classification precision reaching 0.91 at the SNR of 0.05.

The 3D classification precision appears to be strongly correlated with the map quality and resolution homogeneity across the density map (Figure 2E–P and Supplementary Figure S1H). All density maps from AlphaCryo4D consistently show homogeneous local resolutions (at 2.6–2.9 Å for SNR of 0.01) between the NACHT and LRR domains (Figure 2I,M and Supplementary Figures S1H, S2D,J and S3A). By contrast, all density maps by the alternative methods show lower average resolutions and notably heterogeneous local resolutions, with the NACHT domain exhibiting substantially lower resolution than that of the LRR domain, causing blurred features, broken loops and invisible sidechains in NACHT (Figure 2J–L,N–P and Supplementary Figures S2E–G and S3B–D). Thus, the significantly improved 3D classification accuracy by AlphaCryo4D enables 4D reconstruction of the conformational continuum at the atomic level.

**Figure 4:**
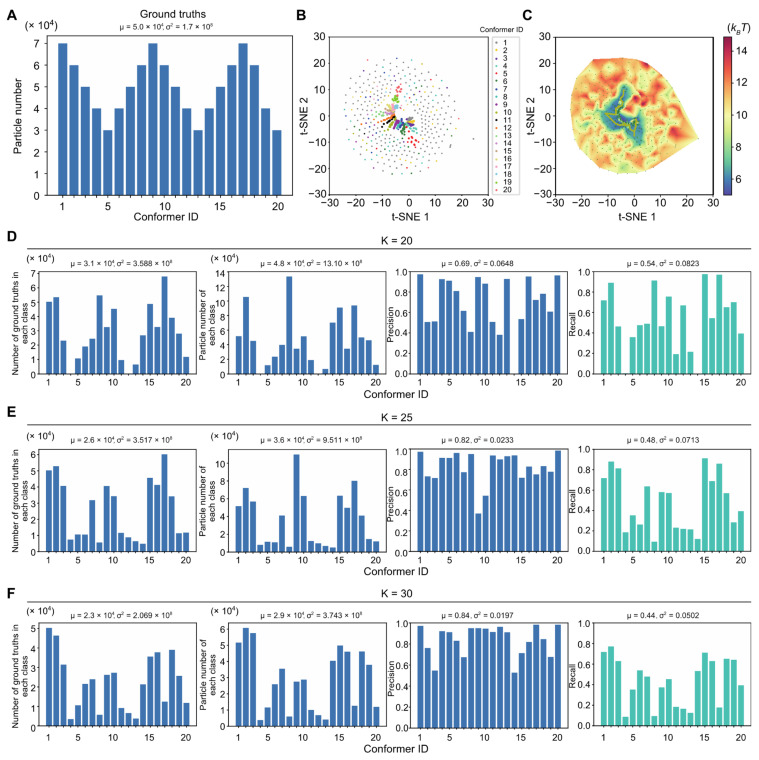
Performance evaluation of 3D classification by AlphaCryo4D on the synthetic dataset with non-uniform distribution of conformational continuum. (**A**) The non-uniform distribution of 20 conformers of another simulated NLRP3 dataset at an SNR of 0.01 was used to examine the performance of AlphaCryo4D. This dataset was utilized to calculate the pseudo-energy landscape shown in panels (**B**,**C**) and its corresponding outcome of 3D classification by AlphaCryo4D shown in panels (**D**–**F**), which tests the robustness of AlphaCryo4D in the case of non-uniform distributions of the underlying conformational states. (**B**) Dimensionality reduction of resampled 3D volumes and their corresponding feature maps from the simulated dataset by t-SNE. The colors of data points indicate the ground truth of their corresponding 3D volumes. (**C**) Reconstruction of the pseudo-energy landscape of the simulated NLRP3 dataset. (**D**–**F**) The number of correctly classified particles in each class, the particle number of each class, the precision of 3D classification and the corresponding recall of AlphaCryo4D blind tests using this simulated dataset when the class number is set to 20 (**D**), 25 (**E**) and 30 (**F**).

Having tested AlphaCryo4D on the simulated datasets with evenly distributed conformers, we further generated a simulated dataset of 0.01 SNR with a non-uniform distribution of the 20 conformers that follows a triangular wave function and presents a more challenging scenario (Figure 4A). The application of AlphaCryo4D on this dataset exhibited only a slight reduction in 3D classification accuracy compared to the results on the datasets of evenly distributed conformers (Figure 4B–D). Increasing the preset class number to 25 or 30 appears to help improve the 3D classification accuracy and bring it back to a level comparable to those obtained on the datasets of evenly distributed conformers (Figure 4E,F). Moreover, the particle distribution computed by AlphaCryo4D appears to approximately recapitulate the triangular wave function (Figure 4D–F), suggesting that AlphaCryo4D is potentially capable of reconstituting the overall statistical distribution of the underlying conformational continuum.

### 2.3. Analysis of Algorithm Mechanism

**Figure 5:**
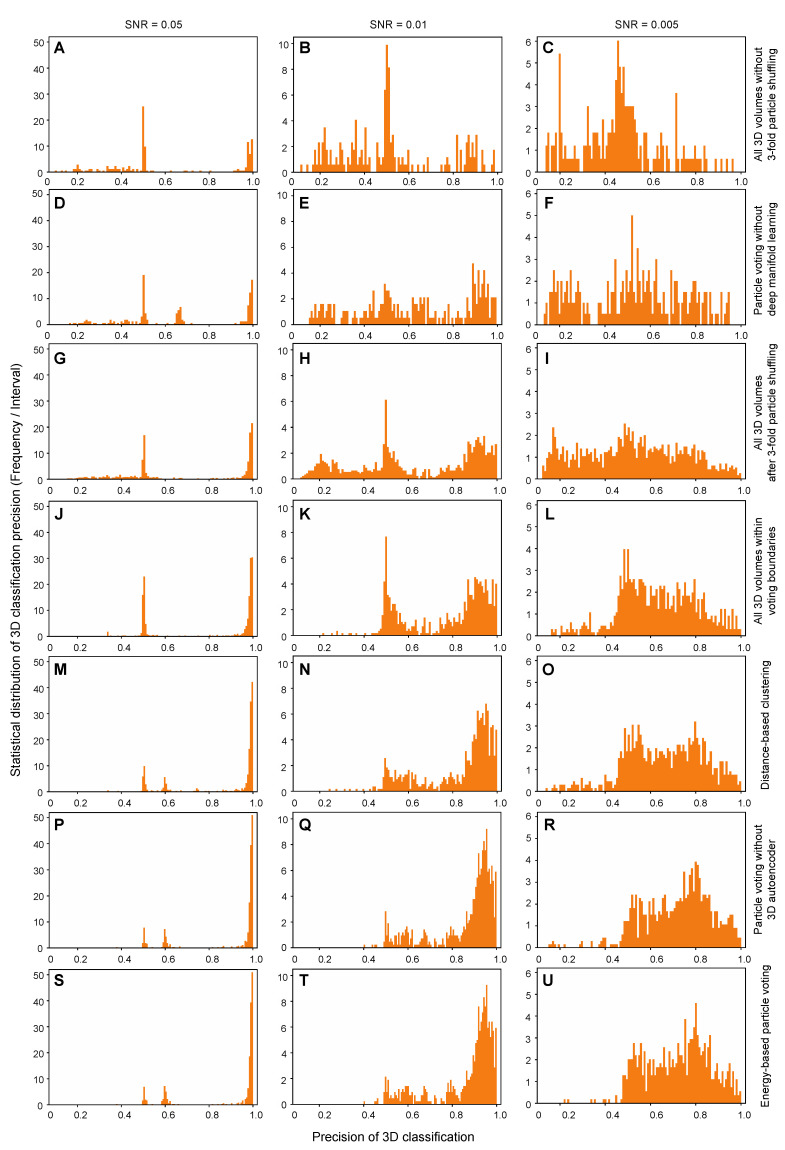
Mechanistic characterizations of the improvement of 3D classification accuracy by AlphaCryo4D using the simulated NLRP3 datasets of three typical SNRs. Left, middle and right vertical columns show the precision analysis on the simulated datasets with SNRs of 0.05, 0.01 and 0.005, respectively. In total, there are 21 conditional controls under six different algorithmic design variations being analyzed in panels (**A**–**R**). (**A**–**C**) Statistical distribution of 3D classification precision in resampled 3D volumes without particle shuffling. (**D**–**F**) Statistical distribution of 3D classification precision by implementing particle voting directly on the resampled 3D volumes without using deep manifold learning. The procedure of 3-fold particle shuffling and volume resampling is identical to AlphaCryo4D. (**G**–**I**) Distribution of 3D classification precision in resampled 3D volumes after 3-fold particle shuffling in the intermediate step of AlphaCryo4D. (**J**–**L**) Distribution of 3D classification precision in all resampled 3D volumes within voting boundaries on the pseudo-energy landscape in the intermediate step of AlphaCryo4D. (**M**–**O**) Distribution of 3D classification precision after distance-based 3D clustering in the absence of energy-based particle voting with all prior steps identical to AlphaCryo4D. (**P**–**R**) Distribution of 3D classification precision after particle voting by a modified AlphaCryo4D variation only without using deep residual autoencoder in the manifold embedding step. (**S**–**U**) Distribution of 3D classification precision after energy-based particle voting via a complete AlphaCryo4D procedure.

To understand how the 3D classification accuracy is improved, we analyzed the algorithmic mechanism by ablating or replacing certain components of AlphaCryo4D (Figure 5 and Supplementary Figure S1D–G). In total, we conducted 24 conditional tests using 10 variations of algorithmic design by removing or replacing certain components of AlphaCryo4D. First, by removing the entire component of deep manifold learning, the distributions of high-precision 3D classes obtained by *M*-fold particle shuffling and voting alone drop by ~60% relative to that from complete AlphaCryo4D processing (Figure 5D–F). In these ablation experiments, the particle voting was achieved by counting the votes of a particle against the same conformers classified by RELION via computing the Intersection-over-Union (IoU) metric after the step of volume resampling. Similarly, by removing the 3D deep residual autoencoder but keeping the manifold learning by t-SNE for pseudo-energy landscape reconstitution, the distributions of high-precision 3D classes are reduced by ~15% at the SNR of 0.005 (Figure 5P–R). The effect of accuracy degradation is less prominent at a higher SNR (0.05 or 0.01), indicating that the unsupervised feature learning using deep residual autoencoder promotes the tolerance of the algorithm against higher noise levels in the data. Together, these ablation tests suggest that deep manifold learning plays a crucial role in improving 3D classification accuracy with low SNR data.

Next, we replaced t-SNE with four other algorithms in the manifold learning step, including two classic linear dimensionality reduction techniques, principal component analysis (PCA) [13] and multidimensional scaling (MDS) [57], and two nonlinear dimensionality reduction algorithms, isometric mapping (Isomap) [58] and locally linear embedding (LLE) [59]. We applied the four algorithms to reduce the dimensionality of the same sets of resampled volume data. Although being capable of differentiating a portion of ground-truth conformers, the resulting 2D mappings by the four techniques exhibit considerable overlap between adjacent conformers and are incapable of distinguishing all 20 conformers of ground truth, thus inevitably missing many conformers (Supplementary Figure S1D–G). The PCA and Isomap missed approximately half of the ground-truth conformers, whereas MDS and LLE both missed around 70% of the ground-truth conformers. The inferior performance of these techniques is consistent with the original control experiments conducted by the t-SNE developers [53].

Further, we conducted control experiments to evaluate the impact of particle voting on the 3D classification accuracy by replacing the particle voting component with a clustering strategy that directly classifies each particle to the cluster of the nearest clustering center (Figure 1E). In this case, the distributions of high-precision 3D classes are reduced by ~15–30% (Figure 5M–O). The reduction is more prominent at the lower SNR. This indicates that particle voting considerably improves the 3D classification accuracy but is less impactful than the component of deep manifold learning.

Last, by tracking the statistics of classification precisions step by step, we evaluated how the 3D classification accuracy is improved over the intermediate steps of AlphaCryo4D (Figure 5G–I). We found that the steps of particle shuffling, defining cluster boundaries on the pseudo-energy landscapes and energy-based particle voting contribute to ~16%, ~20% and ~40% improvements in the distributions of high-precision 3D classes, respectively. Taken together, these results indicate that all components contribute to the improved performance of AlphaCryo4D in 3D classification toward the atomic level. Neither deep manifold learning nor particle voting alone is sufficient to achieve the present level of 3D classification accuracy.

### 2.4. Visualizing Hidden Dynamics of the 26S Proteasome

The 26S proteasome is one of the most complex, dynamic, heterogeneous holoenzyme types of machinery that regulates nearly all aspects of cell physiology in eukaryotes [25,27,60,61,62]. Visualizing atomic structures of nonequilibrium intermediates or transition states connecting the major states of the functional proteasome in the act of substrate degradation has been less successful [4,6,7,25,60]. To test this possibility, we applied AlphaCryo4D to analyze a large experimental dataset of substrate-engaged human 26S proteasome (EMPIAR-10669), which comprises more than 3 million particles [25]. AlphaCryo4D was able to significantly enrich the number of visualized proteasome conformers to 64 using this dataset, which was previously used to determine the atomic structures of seven proteasome states [25]. As the majority of the states were refined to near-atomic resolution, they allowed us to obtain a more complete picture of the functional dynamics of the proteasome at the atomic level.

To demonstrate that AlphaCryo4D can simultaneously improve 3D classification and resolution of key dynamic features, we examine a representative case among numerous improvements of solving the proteasome dynamics by AlphaCryo4D [26]. Here, we focus on state E_A2_ of the substrate-engaged 26S proteasome, which is the initial state that a substrate-conjugated ubiquitin moiety that binds the deubiquitylating enzyme RPN11 to prepare for substrate engagement and deubiquitylation. In this case, we expected to use AlphaCryo4D to detect if an intermediate substrate binding to RPN11 precedes the deubiquitylation step of the proteasome [25].

**Figure 6:**
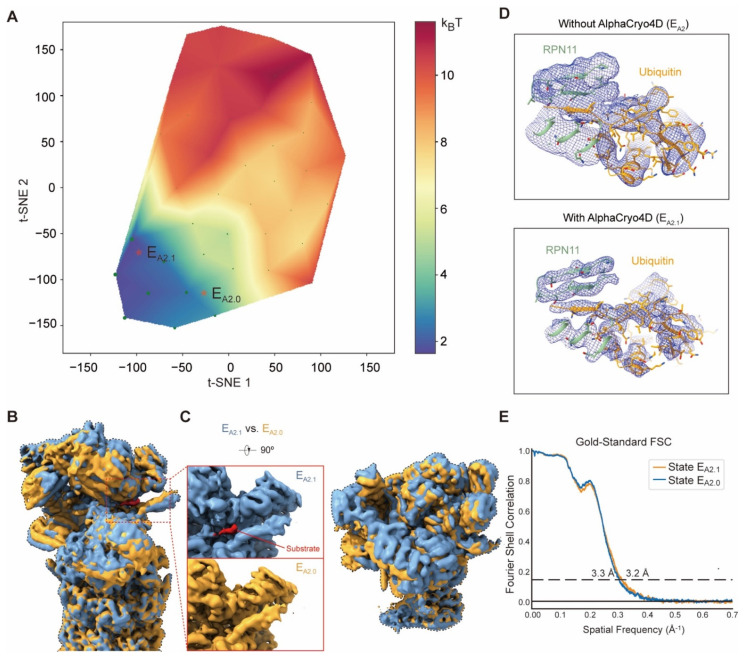
Resolution improvement of the dynamic components of the 26S proteasome by AlphaCryo4D. (**A**) Pseudo-energy landscape calculated on the regulatory particle of the proteasome state E_A2_. Clusters E_A2.0_ and E_A2.1_ were determined on this pseudo-energy landscape, revealing the states of substrate-unbound and substrate-bound RPN11, respectively. (**B**,**C**) Cryo-EM density map of cluster E_A2.0_ superimposed with that of cluster E_A2.1_ from two orthogonal viewing angles. Overall view, panel (**B**). Closeup view, panel (**C**). The lid subcomplex undergoes conformational changes during substrate binding. Insert of panel (**C**) shows a closeup side-by-side comparison of the two states. The red region denoted the substrate in the density map of the 26S proteasome (Supplementary Video S1). (**D**) Quality and resolution improvement of local cryo-EM density map of the RPN11-bound 8.5-kDa ubiquitin in state E_A2.1_ (lower panel) relative to the previously published structure (upper panel) [25]. (**E**) Gold-standard FSC of states E_A2.0_ and E_A2.1_ of 26S proteasome reconstructed by AlphaCryo4D.

The previous reconstruction on state E_A2_ has shown that no substrate but ubiquitin at a moderate resolution was bound to the RPN11 surface [25]. On the pseudo-energy landscape of the sub-dataset corresponding to state E_A2_, we examined two conformational clusters, designated E_A2.0_ and E_A2.1_, which were refined to 3.3 and 3.2 Å, respectively (Figure 6A,E). Comparing these two density maps reveals subtle conformation changes in the lid subcomplex against the base, although both show no substrate binding in the central channel of the hexameric AAA-ATPase ring (Figure 6B). Markedly, state E_A2.1_ exhibits a segment of substrate bound to the cryptic hydrophobic groove at the interface between RPN11 and RPT4 near the active site of RPN11, whereas no substrate is observed at the same site in state E_A2.0_ (Figure 6C). This polypeptide substrate segment has a length of about 4 amino acids, which is comparable in size to many small-molecule compounds, suggesting that AlphaCryo4D may be capable of classifying cryo-EM data toward very small features relevant to drug discovery.

The conformation of state E_A2.0_ appears identical to that of the previously published state E_A2_ [25]. The resolution of the RPN11-bound, 8.5-kDa ubiquitin density in state E_A2_ is around 6–9 Å, although the overall resolution of state E_A2_ was measured at 3.3 Å. Approximately half of the particles previously classified as E_A2_ were also classified as E_A2.1_, indicating the mixture of different conformers that caused the low-resolution feature of ubiquitin in previous studies [25]. Notably, this RPN11-bound ubiquitin in E_A2.1_ clearly shows higher resolution features consistent with 3.8 Å, with sufficiently separated β-strands (Figure 6D). It indicates that the RPT5 N-loop, C-terminal strand of ubiquitin and insert-1 β-hairpin of RPN11 already form a four-stranded β-sheet prior to substrate insertion into the proteasomal AAA-ATPase channel. These high-resolution features allow us to confidently interpret state E_A2.1_ as a transient intermediate between states E_A2_ and E_B_ that were previously reported [25]. Moreover, E_A2.1_ was also observed in the USP14-bound human proteasome in a recent study [63], further verifying this result. Altogether, our extensive application of AlphaCryo4D to the experimental dataset demonstrates its capability in exploring the hidden conformational space of substrate-engaged proteasome at the atomic level, thus providing unprecedented insights into the molecular mechanism of proteasome autoregulation as described in detail elsewhere [26].

### 2.5. Revealing Hidden Conformational Heterogeneity in the Pf80S Ribosome

**Figure 7:**
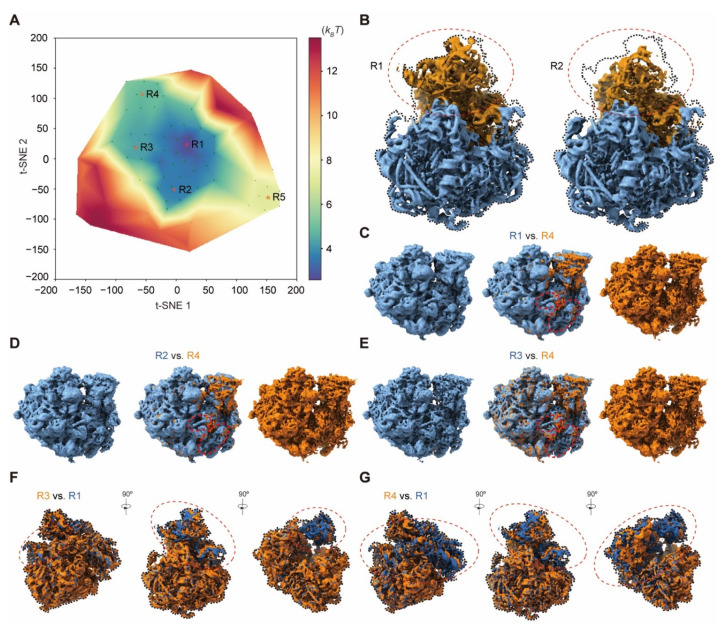
Visualizing hidden conformational dynamics of the *Pf*80S ribosome bound to the anti-protozoan drug at high resolution by AlphaCryo4D. (**A**) Clusters R1-R5 on the pseudo-energy landscape by AlphaCryo4D. Energy-based particle voting was conducted within each cluster to keep particles of high reproducibility. (**B**) Comparison of the density maps of clusters R1 and R2. Cluster R1 is the major state in the *Pf*80S ribosome dataset, while cluster R2 accounts for the state missing the head of 40S (red circles). The dynamic region of the density map is colored orange and the stable region is colored blue. (**C**–**E**) Comparisons of states R1 and R4 (**C**), states R2 and R4 (**D**) and states R3 and R4 (**E**) reveal the differential appearance of the C-terminal helix of eL8 and an rRNA helix. Note that the rRNA helix is only partially presented in state R3. (**F**) State R3 superimposed with state R1 from three different viewing angles. The density of the 40S head region (red circles) rotated relative to the 60S. (**G**) State R4 superimposed with state R1 from three different viewing angles. The density of the whole 40S (red circles) is rotated relative to the 60S (Supplementary Video S2).

We further evaluated the capability of AlphaCryo4D in detecting residual heterogeneity in a mostly homogeneous dataset of the *Plasmodium falciparum* 80S (*Pf*80S) ribosome bound to anti-protozoan drug emetine (EMPIAR-10028) [48]. The original cryo-EM analysis of the *Pf*80S ribosome dataset (105,247 particles) determined a major conformational state and detected flexibility in the head region of the 40S small subunit, which was originally unsolved [48]. Application of AlphaCryo4D to analyze this dataset reveals notable rotation of the 40S in the *Pf*80S ribosome. By reconstitution of the approximately continuous pseudo-energy landscape using 79 density maps, we simultaneously identified two 40S-rotated states (R3 and R4) and another state (R2) with a missing 40S head in addition to the original conformational state (R1) located in the deepest energy well (Figure 7A,B). Notably, AlphaCryo4D was able to maintain the original resolution of the major state at 3.2 Å, while allowing the three new states R2, R3 and R4 to be refined to 3.6, 4.1 and 4.6 Å, respectively, without using more data (Supplementary Figure S5).

On the pseudo-energy landscape, the distance between states R4 and R1 is longer than that between states R3 and R1, indicating that a larger conformational change occurs in state R4. Indeed, superimposing the density maps of states R3 and R1 together reveals that the rotation of 40S is localized within its head region with concerted movement in the central protuberance of 60S, whereas the rest of the 40S exhibits no obvious movement (Figure 7F). By contrast, the entire 40S rotates above the 60S in state R4 relative to R1, and this coincides with the intrinsic structural rearrangement within 60S near the central protuberance and L1 stalk, which appears to also drive the 40S translation relative to the 60S (Figure 7G), indicating a different mode of intersubunit rotation.

Moreover, the reconstructions of R3 and R4 also identified differential rearrangements of small structural elements, including the stepwise disappearance of an rRNA helix and of the intersubunit bridge formed by the eL8 C-terminal helix, in line with previous studies on the *Pf*80S dynamics during translation (Figure 7C–E) [64]. Our analysis suggests that the superficially simple rotation of the 40S subunit is a manifestation of complicated conformational dynamics involving the entire *Pf*80S ribosome. Given that the *Pf*80S ribosome was bound to the anti-protozoan drug visible in all new states (Supplementary Figure S5E), the AlphaCryo4D-enabled analysis of structural dynamics at high resolution opens the door to investigating the drug-modulated allosteric effect relevant to structure-based therapeutic development [48].

### 2.6. Visualizing Conformational Continuum of the Yeast Pre-Catalytic Spliceosome

**Figure 8:**
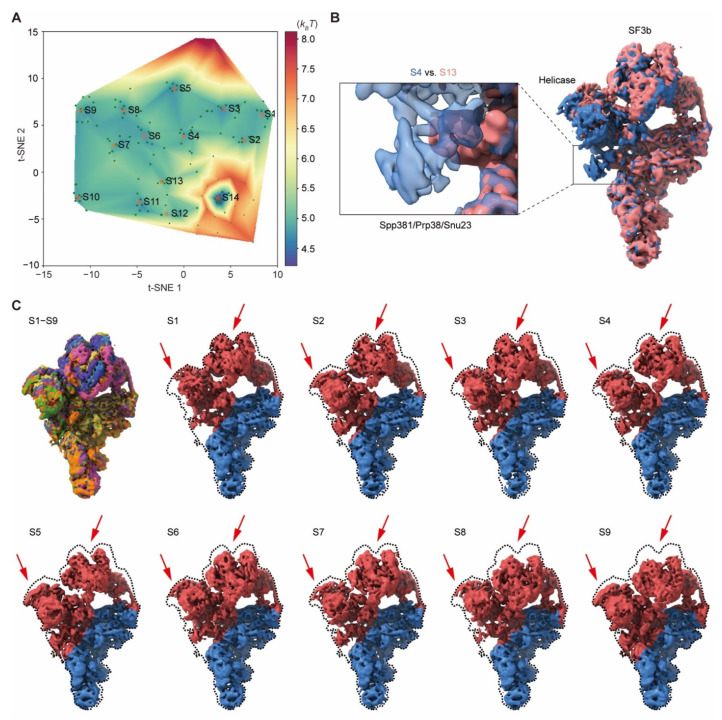
Choreographing continuous inter-subunit motions of the yeast pre-catalytic spliceosome by AlphaCryo4D. (**A**) Pseudo-energy landscape by AlphaCryo4D uncovering 14 clusters designated as S1–S14. Clusters S1–S9 are related to the continuous rotational motion of the SF3b and helicase subcomplexes of the pre-catalytic spliceosome, and the density maps of clusters S10–S14 are shown in Supplementary Figure S6B. (**B**) The density map of the Spp381/Prp38/Snu23-missing state S13 is superimposed with the density map of the state S4. The left inset shows a closeup comparison of the density difference between states S13 and S4 around the subcomplex of Spp381/Prp38/Snu23. (**C**) Cryo-EM density maps of clusters S1–S9. The top leftmost panel shows the superposition of all nine density maps of S1 to S9. In the rest panels, the dynamic component of the density map is colored red and the static component is colored blue. The red arrows highlight the rotational and translational motions of the SF3b and helicase subcomplexes within the dashed outline. The approximate continuous motion of the SF3b and helicase is illustrated in Supplementary Video S3.

We next evaluated the applicability of AlphaCryo4D in simultaneously visualizing continuous motion and discrete compositional heterogeneity in the yeast *Saccharomyces cerevisiae* pre-catalytic spliceosome (EMPIAR-10180) [49]. A previous study using focused classification has provided a multistate-averaged map of the pre-catalytic B complex spliceosome and speculated that the SF3b subcomplex might sample a continuum of conformations [49]. To characterize the pre-catalytic spliceosome dynamics, we resampled 160 density maps using 327,490 particles to reconstitute its pseudo-energy landscape (Figure 8A), which allowed us to determine many intermediate states (S1–S9) representative of a continuous motion of the SF3b and helicase subcomplexes as well as several compositionally distinct states (S10–S14). 

On the pseudo-energy landscape, the nine states S1–S9 representing the continuous motions are intuitively located in adjacent clusters, forming a major MEP traversing the long axis of the pseudo-energy landscape (Figure 8A,C). These states show both independent movements of the SF3b and helicase, as well as concerted motions of the two subcomplexes (Supplementary Figure S6A). For instance, a comparison between states S3 and S4 exhibits that the SF3b and helicase move away from each other, whereas states S5 and S6 show co-directional motions of both the SF3b and helicase. Interestingly, the Cus1 subunit in the SF3b subcomplex appears to swing at the interface with the N-terminal helicase cassettes of RecA-1 and RecA-2, implicating that part of conformational rearrangement is mediated by the dynamic interface B between the SF3b and helicase [49].

In addition to mapping the continuous inter-subunit motion, AlphaCryo4D also reveals the potential allosteric effect of local structural variation relevant to spliceosome activation. Notably, a comparison between states S4 and S13 exhibits that the U2 snRNP and helicase motions among these states appear to be allosterically coupled to the disappearance of the Spp381, Prp38 and Snu23 densities in state S13 (Figure 8B). As the proteins Spp381, Prp38 and Snu23 are required for spliceosome activation [49], these coexisting conformers may be important to understanding the intermediate steps of spliceosome activation. These biologically important dynamic features have not been previously reported from the same dataset [49]. Taken together, our analysis demonstrates that AlphaCryo4D is capable of characterizing continuous conformational changes on a dataset with a moderate size.

### 2.7. Discovery of Hidden Assembly Intermediates of Bacterial Ribosome

**Figure 9:**
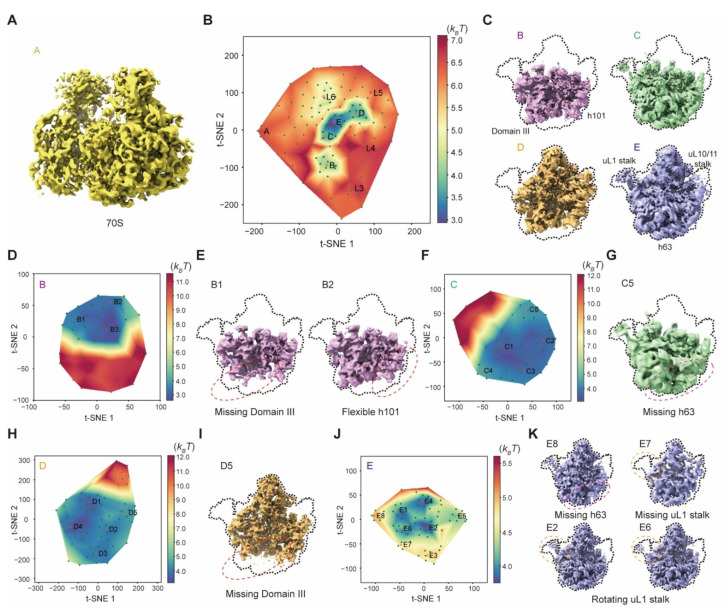
Exploring hidden conformational space of the bacterial ribosomal assembly intermediates by AlphaCryo4D. (**A**) Cryo-EM density map of state A determined by AlphaCryo4D. This state is located far from other states on the pseudo-energy landscape shown in (**B**). (**B**) Global pseudo-energy landscape of bacterial ribosomal assembly intermediates. Clusters A–E and L3–L6 were reconstructed for further analysis. (**C**) Density maps of the major states B, C, D and E were reconstructed by AlphaCryo4D. The density maps of these four states were refined using the particles of clusters B, C, D and E, respectively, from the pseudo-energy landscape. (**D**) Zoomed-in pseudo-energy landscape of the major state B. (**E**) New subclass conformers B1 and B2 of state B. Comparing to the original state B (Supplementary Figure S7B), the new states B1 and B2 appear to miss Domain III and show flexible rRNA helix 101, respectively, as discovered by AlphaCryo4D. (**F**) Zoomed-in pseudo-energy landscape of the major state C generating clusters C1–C5. (**G**) A new state (C5) of C is determined by AlphaCryo4D. The rRNA helix 63 was not observed in the density map of C5. (**H**) Clusters D1–D5 from the zoom-in pseudo-energy landscape of the major state D. (**I**) A newly discovered minor state D5. Like the minor state B1 of the major state B, Domain III was not seen in the density map of D5. (**J**) Clusters E1–E8 on the zoom-in pseudo-energy landscape of the major state E by AlphaCryo4D. (**K**) Discovery of new subclass conformers in the major state E. Like C5, the new state E8 lacks the rRNA helix 63. Compared to the previously reported state E3 (Supplementary Figure S7E), the uL1 stalk is not found in subclass E7. Two new states, E2 and E6, with different conformational changes of the uL1 stalk were discovered by AlphaCryo4D (Supplementary Video S4).

In the last application, we used AlphaCryo4D to analyze a highly heterogeneous dataset of the *Escherichia coli* 50S large ribosomal subunit (LSU) (EMPIAR-10076) [50]. This dataset (131,899 particles) is known to be both compositionally heterogeneous and conformationally dynamic in that the 50S LSU undergoes bL17-depleted intermediate assembly. We first computed the overall pseudo-energy landscape of the ribosomal assembly using 119 density maps (Figure 9B). On this pseudo-energy landscape, the originally reported five major states A–E were straightforwardly reproduced by energy-based particle voting (Figure 9A–C). Obviously, state A containing both 50S and 30S subcomplexes is a rare state distinct from other conformers (Figure 9A). The density map of state A reconstructed by only 766 particles still retains enough details, benefiting from the strategy of energy-based particle voting.

As the remaining particle numbers of states B, C, D and E were all adequate for deeper classification, we then computed their zoomed-in pseudo-energy landscapes separately. This allowed us to discover seven new conformational states (designated B1, B2, C5, D5, E6, E7, E8) that have evaded all other methods (Figure 9D–K) [11,12,19,50], as well as all 13 previously reported sub-class conformers classified from this dataset (Supplementary Figure S7) [12,50]. On the zoomed-in pseudo-energy landscape of state B, state B1 exhibits a low occupancy of domain III as well as another new state D5 that is perhaps the least mature LSU intermediate on the assembly pathway (Figure 9E,I). The rRNA helix 101 exhibits a small rotation between states B2 and B3 (Figure 9E and Supplementary Figure S7B). The differential occupancy of rRNA helix 63 and the movement of the uL1 stalk are observed in states C5 and E6–E8 (Figure 9G,K). Among the sub-states derived from state E, the new state (E7) with uL1 missing was reconstructed by only 357 particles (Figure 9K), which was another rare state discovered via AlphaCryo4D that is only 0.3% of the entire dataset. These results suggest that AlphaCryo4D is highly efficient in finding transient states of extremely low abundance and in exploring the ‘dark’ conformers in the conformational space of highly heterogeneous systems.

## 3. Discussion

One primary objective of the AlphaCryo4D development is to simultaneously improve reconstruction resolution and 3D classification accuracy without assumption on sample behaviors, such as conformational heterogeneity, continuity, or chemical composition. Importantly, AlphaCryo4D has recently been used to improve the state-resolving capacity of time-resolved cryo-EM analysis to yield more than a dozen intermediate conformers of USP14-regulated human proteasomes at atomic detail [63]. Because time-resolved cryo-EM is often conducted on samples flash-frozen in the course of biochemical reactions, such samples often contain a much greater degree of compositional and conformational heterogeneity that may be intuitively pertinent to the functional, lowly populated intermediate states of the imaged systems. Thus, time-resolved cryo-EM in principle requires higher 3D classification accuracy to disentangle many coexisting, low-abundance conformers toward a high enough resolution [63]. This is a major aspect limiting the general application of time-resolved cryo-EM, in which it is often a formidable challenge to make high-resolution sense of time-resolved datasets. Thus, combining a method of high accuracy in 3D classification with time-resolved cryo-EM may be practically desirable and present an emerging paradigm in visualizing functional complex dynamics [63]. Several attempts have been made recently to characterize the so-called ‘continuous motion’ of protein complexes using cryo-EM data [11,12,19,20,21,22]. Nonetheless, it remains hypothetical that the conformational space of protein complex dynamics must be continuous. This may be approximately sensible in a classic picture of molecular mechanics but is not necessarily appropriate in the theoretical framework of quantum mechanics. Thus, a structural ensemble discovered by AlphaCryo4D is preferentially regarded as discrete approximations representing the conformational space of fundamentally unknown continuity.

This study further substantiates the notion that the improved accuracy of 3D classification is positively correlated with the resolution gain because misclassification reduces achievable resolution for reconstructing highly dynamic conformations. Because of extremely low SNR, most objective functions in image similarity measurement and machine learning algorithms tend to poorly perform or entirely fail on cryo-EM data. To date, no methods have included any implicit metrics or strategies for validating 3D classification accuracy in cryo-EM. We address this issue by introducing the *M*-fold particle shuffling method and the energy-based particle voting algorithm in AlphaCryo4D that automatically checks the reproducibility and robustness of the 3D classification. This allows users to objectively reject non-reproducible particles and permits a maximal number of particles to be assessed and classified in an integrative procedure based on uniform, objective criteria. Our extensive tests suggest that the particle-voting algorithm synergistically enhances the performance of deep manifold learning. Moreover, it can potentially rescue certain particles that are prone to be misclassified if processed only once by deep manifold learning, thus boosting the efficiency of particle usage without necessarily sacrificing the quality and homogeneity of selected particles. This mechanism simultaneously optimizes the usage of all available particles and their 3D classification accuracy for achieving higher resolution.

Our particle-shuffling and Bayesian resampling strategy are conceptually distinct from the stochastic bootstrap method previously proposed for estimating 3D variance maps [65] or performing 3D PCA [13] to detect conformational variability. One essential difference lies in that our resampling approach requires that only particles of similar or identical conformations are grouped together via likelihood-based similarity estimation instead of being combined or resampled in a stochastic manner [1,18]. Another difference is that the goal of *M*-fold particle shuffling is to improve the quality of the reconstituted pseudo-energy landscape and to enable energy-based particle voting for the intrinsic cross-validation of 3D classification. Furthermore, AlphaCryo4D does not assume any prior knowledge regarding the conformational landscape, its continuity and topology, as well as chemical composition of the macromolecular system. Our approach allows multiple consensus models being used for optimizing the particle alignment during Bayesian resampling. This can theoretically deal with more complicated conformational dynamics when no stable core structure is available to guide the consensus alignment over a single model.

Using a pseudo-energy landscape to represent the conformational variation has theoretical roots in the physical chemistry of protein dynamics. Extensive research tools being built upon the energy landscape and transition-state theory allow comparison with other complementary approaches, such as single-molecule florescence microscopy, and nuclear magnetic resonance (NMR) and molecular dynamics (MD) simulation. Particularly, all-atom MD simulation with enhanced sampling techniques allows for the computation of the virtual energy landscape of kilodalton biomolecular systems over a limited timescale often up to a millisecond [66,67]. Due to the limited capacity of supercomputing resources, this approach is not yet commonly feasible for recapitulating large complex conformational changes of megadalton biomolecular systems in their functional actions. By contrast, AlphaCryo4D reconstitutes pseudo-energy landscape from experimental datasets of megadalton molecular weight, which may sample significantly larger conformational space over a functional course of much longer timescale up to minutes and even hours, when it is used in conjunction with time-resolved cryo-EM [63]. With the continuous growth of supercomputing capacity, it might become possible in the future for the two approaches to be compared directly and cross-validated for the results from each other, given certain caveats being adequately dealt with, such as the appropriate calibration of energy scale and authentic estimation of energy resolution. Alternatively, part of the enhanced sampling techniques, such as the string method, has been integrated into the framework of AlphaCryo4D. Thus, it should be feasible to borrow other enhanced sampling techniques in the future development of AlphaCryo4D [66,67].

However, the primary goal of mapping pseudo-energy landscape in AlphaCryo4D is to improve the accuracy of 3D classification rather than the pursuit of the authenticity of characterizing free-energy landscape, owing to a limited energy resolution and non-intuitive dimensionality reduction used for the mapping procedure. In addition, the pseudo-energy landscape allows users to qualitatively examine kinetic relationships between the adjacent conformers and potentially discover new conformations. Quantitatively deriving biochemical kinetics from such a pseudo-energy landscape is not advised and may be error-prone. We stress that the reconstitution of an authentic free-energy landscape from cryo-EM data remains an open question rather than a solved one. While our method of mapping 3D volumes to manifolds increases the sampling grain in approximating the conformational space as compared to other methods directly mapping single particles to the latent space [11,12,19], AlphaCryo4D trades off the fineness of sampling grain in visualizing conformational space for resolution gain. In nearly all cases we examined, AlphaCryo4D apparently still oversampled the conformational space at large in terms of finding those conformational states that can potentially achieve high resolution.

The key steps of AlphaCryo4D processing add about 20% of computational costs in addition to those commonly practiced steps, including initial consensus alignment and final high-resolution refinement (see Section 4.15 below). We speculate that larger datasets are generally required to fully exploit the advantages and potentials of cryo-EM in solving heterogeneous structures, no matter which approach is used. The data size is expected to proportionally scale with and match the degree of conformational heterogeneity to visualize a full spectrum of conformational states at the atomic level. Thus, further optimization of the algorithmic design and code-executing efficiency via software engineering will help to reduce the computational costs and to make AlphaCryo4D more affordable for widespread applications.

Several limitations of AlphaCryo4D are in order. First, its outcomes depend on the success of the initial consensus alignment of all particles during particle shuffling and volume resampling. Certain conformational dynamics or heterogeneity can interfere with image alignment, in which case the performance of AlphaCryo4D will be restricted by alignment errors after consensus refinement. This is a common problem for all existing methods developed so far. Our tentative solution is to improve image alignment in a multi-reference refinement procedure. Failure in obtaining accurate alignment parameters can lead to futile, erroneous estimation of conformational heterogeneity in the subsequent steps no matter which methods are used.

Second, the use of t-SNE to visualize a pseudo-energy landscape poses both potential advantages and limitations. The t-SNE algorithm can well preserve the local topology of a pseudo-energy landscape but is not warranted to preserve global topology, which is nonetheless a common limitation for existing manifold learning techniques. The geometry of the estimated pseudo-energy landscape is inevitably influenced by the SNRs of cryo-EM data; lower SNRs cause dispersion of local energy wells and thus the overall geometrical distortion of the landscape (Supplementary Figure S1). A higher level of noise introduces greater statistical bias in particle clustering into resampled 3D volumes and causes irreproducible particle ‘votes’. To alleviate this issue, the particle-voting algorithm is employed in the final 3D classification of the local areas of energy minima on a pseudo-energy landscape, which rejects particles with irreproducible votes.

Last, all existing approaches for visualizing a pseudo-energy landscape from cryo-EM data suffer from the ‘curse of dimensionality’, because the dimensions of reaction coordinates related to the conformational variation are expected to be very high in real space for a megadalton macromolecular complex that includes hundreds of thousands of atoms [40,41,42,43,44,45,46]. Thus, the reduction of dimensions prior to the visualization of a pseudo-energy landscape is necessary. The reduced dimensions then lose intuitive physical meanings that differ from case to case. In all cases of experimental and simulated datasets in this study, the reduced dimensions by t-SNE appear to capture the most prominent conformational changes, analogous to the principal components computed by PCA. Thus, dimensionality reduction to 2D by t-SNE, as well as MEP searching on a 2D pseudo-energy landscape might limit its capability in disentangling complex dynamics governed by many reaction coordinates. Further investigations are required to understand how to map individual particles to pseudo-energy landscape without trading off reconstruction resolution, to estimate pseudo-energy landscape at higher dimensions, to preserve its global topology with high fidelity and to fully automatize MEP solution.

In summary, the development of AlphaCryo4D and its applications in several important biomolecular systems have demonstrated the feasibility of atomic-level visualization of conformational space hidden in a non-equilibrium regime or corresponding to transient transitions between different metastable states, particularly for biomolecular systems that are too large or complex to be studied by all-atom MD simulations. Applications of this approach to important therapeutic targets in the future might unlock atomic views of cryptic or mobile sites for ligand discovery that otherwise remain invisible to alternative techniques. Our studies of AlphaCryo4D provide essential mechanistic insights into the computational problem of efficiently extracting atomic-level dynamics information from cryo-EM data. We expect that AlphaCryo4D will become an important steppingstone and inspire more efforts in leveraging machine learning algorithms to develop the next-generation cryo-EM pipelines that overcome the aforementioned limitations and push the envelope of visualizing functional dynamics of nonequilibrium biomolecular systems at the atomic level.

## 4. Materials and Methods

### 4.1. M-Fold Particle Shuffling and Bayesian Resampling

A key philosophy of the AlphaCryo4D design is to avoid subjective judgment on the particle quality and usability if it is not apparent false particles, such as ice contaminants, carbon edges and other obvious impurities. Deep-learning-based particle picking in DeepEM v1.0 (Peking University, Beijing, China) [68] or other similarly performed software is favored for data preprocessing prior to AlphaCryo4D. To prepare particle datasets for AlphaCryo4D, an initial unsupervised 2D image classification and particle selection, preferentially conducted by the statistical manifold-learning-based algorithm in ROME v1.1.2 (Peking University, Beijing, China) [5], is necessary to ensure that no apparent false particles are selected for further analysis and that the data have been collected under an optimal microscope alignment condition, such as optimized coma-free alignment. No particles should be discarded based on their structural appearance during this step if they are not apparent false particles. Any additional 3D classification should be avoided to pre-maturely reject particles prior to particle shuffling and volume resampling in the first step of AlphaCryo4D processing. Pre-maturely rejecting true particles via any 2D and 3D classification is expected to introduce subjective bias and impair the native conformational continuity and statistical integrity intrinsically existing in the dataset.

In raw cryo-EM data, 2D transmission images of biological macromolecules suffer from extremely heavy background noise, due to the use of low electron dose to avoid radiation damage. To tackle the conformational heterogeneity of the macromolecule sample of interest in the presence of heavy image noise, the particle shuffling and volume resampling procedure was devised to incorporate the Bayesian or maximum-likelihood-based 3D clustering in RELION v2.1 or v3.0 (MRC, Cambridge, UK) [3]. To enable the particle-voting algorithm in the late stage of AlphaCryo4D, each particle is reused *M* times during particle shuffling to resample a large number of 3D volumes (Figure 1B). First, all particle images are aligned to the same frame of reference in a consensus 3D reconstruction and refinement in RELION [3] or ROME [5] to obtain the initial alignment parameters of three Euler angles and two translational shifts. Optimization for alignment accuracy should be pursued to avoid the error propagation to the subsequent steps in AlphaCryo4D. For a dataset with both compositional and conformational heterogeneity, coarsely classifying the dataset to a few 3D classes during initial image alignment, limiting the 3D alignment to a moderate resolution, such as 10 Å or 15 Å during a global orientational search, progressing to small enough angular steps in the final stage of consensus refinement, may be optionally exercised to optimize the initial 3D alignment. Failure of initial alignment of particles would lead to failure of all subsequent AlphaCryo4D analyses.

Next, based on the results of consensus alignment, in the particle-shuffling step, all particles were divided into *M* + 1 groups, and *M* was set to an odd number of at least 3. Then the whole dataset was shuffled *M* + 1 times by removing a different group from the dataset each time. Each shuffled dataset is classified into a large number (*B*) of 3D volumes, often tens to hundreds, by the maximum-likelihood 3D classification algorithm without further image alignment in RELION (with ‘skip-align’ option turned on). This is necessary because the alignment accuracy often degrades when the sizes of 3D classes decrease considerably. This step is repeated *M* + 1 times, each time on a shuffled dataset missing a different group among the *M* + 1 groups. Because of *M*-fold particle shuffling, the outcome of this entire process is expected to resample up to thousands of 3D volumes in total (i.e., *B* (*M* + 1) > 1000). Each particle is used and contributed to the 3D reconstructions of *M* volumes, which prepare it for the particle-voting algorithm to evaluate the robustness and reproducibility of each particle with respect to the eventual 3D classification.

For processing a large dataset including millions of single-particle images, it becomes infeasible even for a modern high-performance computing system to do the consensus alignment by including all particles once in a single run due to the limitations of supercomputer memory and the scalability of the alignment software. To tackle this issue, the whole dataset is randomly split into a number (*D*) of sub-datasets for batch processing, with each sub-dataset including about one to two hundred thousand particles, depending on the scale of the available supercomputing system. In this case, the initial reference should be used for the consensus alignment of different sub-datasets to minimize volume alignment errors in the later step. The total number of resulting resampled volumes becomes *BD* (*M* + 1) > 1000. This strategy can substantially reduce the supercomputer memory pressure and requirement. In each sub-dataset, all particles were divided into *M* + 1 groups and subject to the particle shuffling and volume resampling procedure as described above.

### 4.2. Deep Residual Autoencoder for 3D Feature Extraction

The resampled volumes may still suffer from reconstruction noises and errors due to variation of particle number, misclassification of conformers and limited alignment accuracy. Thus, we hypothesize that unsupervised deep learning could help capture the key features of structural variations in the resampled volumes and potentially enhance the quality of subsequently reconstituted pseudo-energy landscapes for improved 3D classification. To this end, a 3D autoencoder was constructed using a deep Fully Convolutional Network (FCN) composed of residual learning blocks [51,52]. The architecture of the 3D autoencoder consists of the encoder and the decoder, which are denoted as ℰ and D, respectively (Figure 1C). The relation between the output y and the input x of the network can be expressed as y=D(ℰ(x)), in which x is the input 3D density volume with the size of *N*^3^, where *N* is the box size of the density map in pixel units. For reconstruction of the 3D volumes and further optimization, the decoding output y should be in the same size and range with the input data x. In this way, the framework of FCN is established to restore the input volume, using the sigmoid function S(x)=1/(1+exp(−x)) as the activation function of the decoding layer to normalize the value of y into the range (0, 1). Meanwhile, all 3D density maps x are normalized with the following equation before input to the deep neural network:

(1) $$x_{ijk}∶=\frac{x_{ijk}-x_{min}}{x_{max}-x_{min}},\;\;\;i,j,k=1,2,\ldots ,N.$$

where $x_{min}$ and $x_{max}$ are, respectively, the minimum and minimax value in all $x_{ijk}$.

The distance between the decoded maps and the input volumes can be used for constructing the loss function to train the 3D kernels and bias of the networks. The value distribution of the encoded 3D feature maps z=ℰ(x) is expected to be an abstract, numerical representation of the underlying structures in the volume data, which may not necessarily have any intuitive real-space physical meanings. The neural network can extract such abstract information in the prediction step, with no restriction on the feature maps z in the expression of training loss. The loss function is then formulated as:

(2) $$L\left(\theta ;x,y\right)=\frac{1}{N^{3}}\overset{N}{\underset{i,j,k=1}{\sum }}\|x_{ijk}-y_{ijk}{\|}^{2}+\lambda \|\theta {\|}^{2}$$

where θ denotes the weights and bias of the network, and λ is the L2 norm regularization coefficient. As the feature of the complex structure is difficult to be learned from the 3D volume data, the value of λ is set to 0.001 in default to prevent overfitting in the applications to experimental datasets. However, it is set to 0 in the tests on the simulated datasets (see below).

To improve the learning capacity of the 3D autoencoder, residual learning blocks containing 3D convolutional and transposed convolutional layers are employed in the encoder and decoder, respectively. In each residual learning block, a convolutional layer followed by a Batch Normalization (BN) [69] layer and an activation layer appears twice as a basic mapping, which is added with the input to generate the output. The mathematical expression of the lth block can be shown as:

(3) $$\{\begin{array}{c} y_{l}=ℱ\left(x_{l};\theta_{l}\right)+x_{l}\\ ℱ\left(x_{l};\theta_{l}\right)=C\left(C\left(x_{l}\right)\right)\\ C\left(x_{l}\right)=a\left(b\left(c\left(x_{l}\right)\right)\right) \end{array}\;$$

where $x_{l}$ and $y_{l}$ represent the input and the output of the block, respectively. $ℱ\left(x_{l};\theta_{l}\right)$ denotes the basic mapping of the lth block parameterized by $\theta_{l}$, and $C\left(x_{l}\right)$ is the sequential operation of convolution or transposed convolution c, BN b and activation a. The rectified linear unit (ReLU) function is used in all the activation layers but the last one. In addition, the output of mapping function $ℱ\left(x_{l};\theta_{l}\right)$ must have the same dimension as the input $x_{l}$. If this is not the case, the input $x_{l}$ must be rescaled along with $ℱ\left(x_{l};\theta_{l}\right)$ using a convolutional or transposed convolutional transformation $c^{\prime }\left(x_{l}\right)$ with an appropriate stride value, the parameters of which can be updated in the training step.

### 4.3. Autoencoder Training

To analyze a large number of volumes, we carefully trained the 3D deep residual autoencoder to obtain suitable kernels and biases. First, parallel computation with multiple GPUs has been implemented to reduce the training time. Then the parameters of the network are optimized by the stochastic gradient descent Adam (Adaptive moment estimation) algorithm, in which the gradients of the objective function L(θ;x,y) with respect to the parameters θ can be calculated by the chain rule. Moreover, the learning rate is reduced to one-tenth when the loss function does not decrease in three epochs based on the initial value of 0.01. After training about 50 epochs, the best model is picked to execute the task of structural feature extraction. Using the unsupervised 3D autoencoder, the feature maps z=ℰ(x) encoding the structural discrepancy among the 3D volume data can be extracted automatically without any human intervention.

### 4.4. Autoencoder Hyperparameters

The recommended hyperparameters for the deep residual autoencoder architecture are provided in Table 1. Although we expand the residual network (ResNet) into 3D, we keep the original design rules of ResNet for constructing the 3D autoencoder [51,52]. The first and last convolutional layers have 5 × 5 × 5 filters (kernels). The remaining convolutional layers have 3 × 3 × 3 filters. Because the cubic filter in the convolutional layer is computationally expensive, only one 3D filter is used in each of the last three convolutional layers in the encoder and of the first two and last transposed convolutional layers in the decoder. To accommodate a large volume input, two filters are used in the first three convolutional layers and in the third and fourth transposed convolutional layers. Downsampling is directly performed by convolutional layers with a stride of 2. The output dimension of encoding layers is set to be a quarter of the input dimension to avoid over-compressing the feature maps. We employed six 3D convolutional layers in the encoder and five transposed convolutional layers in the decoder based on the expected tradeoff between the learning accuracy and training cost, which is roughly comparable to a 2D ResNet with more than 200 layers with respect to the training cost. Lastly, one should randomly inspect some feature maps to ensure that all hyperparameter setting works as expected. If a feature map clearly shows no structural correspondence to its input volume and exhibits strong noise or abnormal features, it might signal the existence of overfitting. In this case, a non-zero value of λ in the L2 norm regularization should be empirically examined to avoid overfitting.

### 4.5. Manifold Embedding of Pseudo-Energy Landscape

To prepare for the pseudo-energy landscape reconstitution, each resampled 3D volume was concatenated with its 3D feature map learned by the 3D autoencoder to form an expanded higher-dimensional data point. Each expanded data point is either low-pass filtered at a given resolution (5 Å in default setting) or standardized by the z-score method prior to t-SNE processing:

(4) $$\{\begin{array}{c} x_{ijk}∶=\frac{x_{ijk}-\mu_{x}}{\sigma_{x}},\;\;\;i,j,k=1,2,\ldots ,N.\\ z_{ijk}∶=\frac{z_{ijk}-\mu_{z}}{\sigma_{z}},\;\;\;i,j,k=1,2,\ldots ,N^{\prime }. \end{array}$$

in which $\mu_{x}$ and $\sigma_{x}$ denote the mean and standard deviation of the 3D volume voxels $x_{ijk}$ with the box size of N, respectively. Similarly, $\mu_{z}$ and $\sigma_{z}$ are the mean and standard deviation of the 3D feature map voxels $z_{ijk}$ with the box size of $N^{\prime }$, respectively. All the input data points were then embedded onto a low-dimensional manifold via the t-SNE algorithm by preserving the geodesic relationships among all high-dimensional data [53]. During manifold embedding, it is assumed that the pairwise similarities in the high dimensional data space and low dimensional latent space follow a Gaussian distribution and a student’s t-distribution, respectively. To find the low-dimensional latent variable of each data point, the relative entropy, also called the Kullback-Leibler (KL) divergence, is used as an objective function measuring the distance between the similarity distribution $p_{ij}$ in the data space and $q_{ij}$ in the latent space:

(5) $$KL\left(P∥Q\right)=\underset{i,j}{\sum }p_{ij}\log\frac{p_{ij}}{q_{ij}}$$

which is minimized by the gradient descent algorithm with the momentum method [70]. The hyperparameter of t-SNE may be tuned to better preserve both local and global geodesic distances (https://distill.pub/2016/misread-tsne/, accessed on 2 August 2022).

The idea of using concatenated data composed of both volumes and their feature maps for manifold embedding is similar to the design philosophy of deep residual learning, in which the input and feature output of a residual learning block are added together to be used as input of the next residual learning block. Such a design has been demonstrated to improve learning accuracy and reduce the performance degradation issues when the neural network goes much deeper [51,52]. Although the 3D feature maps or the resampled volumes alone can be used for manifold embedding, both appear to be inferior in 3D classification accuracy (Figure 5). The concatenated format of input data for manifold learning is potentially beneficial to the applications in those challenging scenarios, such as visualizing a reversibly associated small ubiquitin protein (~8.6 kDa) of low occupancy by the focused AlphaCryo4D classification [26].

After the manifold embedding by t-SNR, each 3D volume is mapped to a low-dimensional data point in the learned manifold. The coordinate system, in which the low-dimensional representation of the manifold is embedded, is used for reconstructing a pseudo-energy landscape. The difference in the Gibbs free energy Δ*G* between two states with classified particle numbers of *N_i_* and *N_j_* is defined by the Boltzmann relation *N_i_*/*N_j_* = exp(−Δ*G*/*k_B_T*). Thus, the free energy of each volume can be estimated using its corresponding particle number:

(6) $$\Delta \Delta G_{i}=-k_{B}T\ln\frac{N_{i}}{\sum_{k}N_{k}}$$

where $\Delta \Delta G_{i}$ denotes the free energy difference of the data point with the classified particle number of $N_{i}$ against a common reference energy level, $k_{B}$ is the Boltzmann constant and T is the temperature in Kelvin. The pseudo-energy landscape was plotted by interpolation of the free energy difference in areas with sparse data. We suggest that linear interpolation is used for the pseudo-energy landscape with loosely sampled areas to avoid overfitting by polynomial or quadratic interpolation. For densely sampled pseudo-energy landscapes, polynomial interpolation could give rise to a smoother pseudo-energy landscape that is easier to be tackled by the string method for MEP solution (see below).

The coordinate system of the embedded manifold output by t-SNE is inherited by the reconstitution of pseudo-energy landscape as reaction coordinates. They do not have an intuitive physical meaning of length scale in real space. However, they can be viewed as transformed, rescaled, reprojected coordinates from the real-space reaction coordinates along which the most prominent structural changes can be observed. Alternatively, they can be intuitively understood as being similar to transformed, reprojected principal components (PCs) in principle component analysis (PCA). In the case of the 26S proteasome, the two most prominent motions are the rotation of the lid relative to the base and the intrinsic motion of the AAA-ATPase motor [25], which can be approximately projected to two reaction coordinates. In real space, both motions are notably complex and in fact are characterized by a considerable number of degrees of freedom, which are partially defined by the solved conformers.

### 4.6. String Method for Finding Minimum Energy Path (MEP)

The string method is an effective algorithm to find the MEP on the potential energy surface [54]. To extract the dynamic information implicated in the experimental pseudo-energy landscape, an improved and simplified version of the string method has been previously developed [55]. Along the MEP on the pseudo-energy landscape, the local minima of interest could be defined as 3D clustering centers to guide the particle-voting algorithm for 3D classification to generate high-resolution cryo-EM density maps (Figure 1D and Figure 2C). The objective of the MEP identification in energy barrier-crossing events lies in finding a curve γ having the same tangent direction as the gradient of energy surface ∇G. It can be expressed as:

(7) $${\left(\nabla G\right)}^{⊥}\left(\gamma \right)=0$$

where ${\left(\nabla G\right)}^{⊥}$ denotes the component of ∇G perpendicular to the path γ. To optimize this objective function, two computational steps referred to as the ‘evolution of the transition path’ and ‘reparameterization of the string’, are iterated until convergence within a given precision threshold.

After initialization with the starting and ending points, in the step of evolution of the transition path, the positions of interval points along the transition path were updated according to the gradient of the free energy at the tth iteration:

(8) $$\varphi_{i}^{*\left(t\right)}=\varphi_{i}^{\left(t-1\right)}-h\nabla G\left(\varphi_{i}^{\left(t-1\right)}\right)$$

with $\varphi_{i}^{\left(t\right)}\;\left(i=0,1,\ldots ,N\right)$ being the ith intermediate point along the transition path at the tth iteration (∗ denoting the temporary values), and h the learning rate.

In the step of reparameterization of the string, the values of positions $\varphi_{i}^{\left(t\right)}\;\left(i=0,1,\ldots ,N\right)$ were interpolated onto a uniform mesh with a constant number of points. Prior to interpolation, the normalized length $\alpha_{i}^{*\left(t\right)}\;\left(i=0,1,\ldots ,N\right)$ along the path was calculated as:

(9) $$\alpha_{0}^{*\left(t\right)}=0,\alpha_{i}^{*\left(t\right)}=\alpha_{i-1}^{*\left(t\right)}+\frac{\left\|\varphi_{i}^{*\left(t\right)}-\varphi_{i-1}^{*\left(t\right)}\right\|}{\sum_{i=1}^{N}\|\varphi_{i}^{*\left(t\right)}-\varphi_{i-1}^{*\left(t\right)}\|},i=1,2,\ldots ,N.$$

Given a set of data points $\left(\alpha_{i}^{*\left(t\right)},\;\varphi_{i}^{*\left(t\right)}\right)$, the linear interpolation function was next used to generate the new values of positions $\varphi_{i}^{\left(t\right)}\;\left(i=0,1,\ldots ,N\right)$ at the uniform grid points $\alpha_{i}^{\left(t\right)}\;\left(i=0,1,\ldots ,N\right)$. The iteration was terminated when the relative difference $\overset{N}{\underset{i=0}{\sum }}\varphi_{i}^{\left(t\right)}-\varphi_{i}^{\left(t-1\right)}{}^{2}/N$ became small enough.

### 4.7. String Method Parameters

The string method of searching for a rational MEP on the pseudo-energy landscape can only guarantee the solution of local optimum and is not capable of ensuring a solution of global optimum [55]. The outcome of the string method depends on the initialization of the starting and ending points of the MEP on the pseudo-energy landscape. If there are too many local minima to sample along a long MEP, the string method could retrieve an MEP solution that partly misses some local energy minima by going off the pathway. In this case, the search for an expected MEP solution can be divided into several segments of shorter MEPs connecting one another, with each MEP being defined by a closer pair of starting and ending points, which travels through a smaller number of energy minima. Another parameter affecting the MEP solution is the step size being set to explore the MEP on pseudo-energy landscapes. Although a default value of 0.1 empirically recommended may work in many cases, it may be helpful to tune the step size according to the quality of the pseudo-energy landscape. The value of step size can be decreased if the computed path runs out of the pseudo-energy landscape and can be increased if the path updates too slowly during iterative searching by the string method.

### 4.8. Energy-Based Particle Voting Algorithm

The particle-voting algorithm was designed to conduct 3D classification, particle quality control, reproducibility test and particle selection in an integrative manner. The particle-voting algorithm mainly involves two steps (Figure 1D). First, we count the number of votes for each particle mapped within each voting boundary. The particles voted for a given voting boundary define a new 3D cluster approximately centered on a local energy minimum of the pseudo-energy landscape. One vote is rigorously mapped to one copy of the particle used in reconstructing a 3D volume and to no more than one 3D cluster on the pseudo-energy landscape where the corresponding volume is located. Thus, each particle can have *M* votes cast for no more than *M* 3D clusters. If the vote is mapped outside of any 3D cluster boundary, it becomes an ‘empty vote’ with no cluster label. Each non-empty vote is thus labeled for both its particle identify and corresponding cluster identity. For each pair of particles and cluster, we compute the total number (*V*) of votes that the cluster receives from the same particle. Each particle is then assigned and classified to the 3D cluster that receives *V* > *M*/2 votes from this particle (Figure 1D). Note that after particle voting, each particle is assigned no more than once to a 3D class, with its redundant particle copies removed from this class. This strategy only retains the particles that can reproducibly vote for a 3D cluster corresponding to a homogeneous conformation, while abandoning those non-reproducible particles with divergent, inconsistent votes.

### 4.9. Alternative Strategy of 3D Clustering without Particle Voting

Because the particle-voting algorithm imposes strong constraints on the reproducibility of particle classification by deep manifold learning, some 3D classes might be assigned with a small number of particles that are insufficient to support high-resolution reconstruction. To remedy this limitation, an alternative, distance-based classification algorithm was devised to replace the particle-voting algorithm when there are not enough particles to gain the advantage of particle voting (Figure 1E). In this method, the distances of all *M* copies of each particle to all 3D cluster centers on the pseudo-energy landscape are measured and ranked. Then, the particle is classified into the 3D cluster of the shortest distance. A threshold could also be manually preset to remove particles that are too far away from any of the cluster centers. The distance-based classification method can keep more particles, but it ignores the potential issue of irreproducibility of low-quality particles. Thus, it is proven to be less accurate in 3D classification (Figure 5M–O). In other words, it trades off the classification accuracy and class homogeneity to gain more particles, which is expected to be potentially useful for small datasets or small classes. By contrast, the energy-based particle-voting algorithm imposes a more stringent constraint to select particles of high reproducibility during classification, resulting in higher quality and homogeneity in the classified particles, which is superior to the distance-based classification method (Figure 5S–U).

### 4.10. Practical Consideration of Particle Shuffling and Voting Parameters

The parameter *M* determines the degree of implicit cross-validation of classification reproducibility, as well as the sampling densities of the pseudo-energy landscape. To establish reproducibility for 3D classification, *M* should be no less than 3. The variation of *M* is not supposed to change the pseudo-energy landscape because it multiplies on both denominator and enumerator in the Boltzmann relation and is canceled in the ratio of particle densities in computing the free-energy differences. Increasing the *M* value will allow more volumes to be resampled, which then leads to a higher sampling density in computing the manifold of the pseudo-energy landscape and potentially enhances its reconstitution quality. The default classification threshold *M*/2 is the minimal number of votes for verifying the reproducibility of 3D classification. However, a higher threshold, such as 2*M*/3, will give rise to more stringent criteria in cross-validation, with a tradeoff of voting out more particles. The particle voting algorithm does not entirely eradicate misclassified particles. However, increasing either *M* or the classification threshold could theoretically have a similar impact and improve the conformational homogeneity, because it gets less probable for a particle to be misclassified to the same cluster more times.

Several considerations may be applied to the choice of *M* and to set up particle shuffling and voting. First, to obtain a high-quality pseudo-energy landscape, a thousand or more resampled volumes are expected for datasets of more than 1–2 million particles. Second, the average particle number per volume is expected to be no less than 5000 or more to ensure that majority of volumes include sufficient particles for quality reconstructions. The expected particle number per volume must increase if the average SNR per particle is decreased. It may need to reach 10,000–20,000 or more for small proteins or lower SNR datasets. Third, for a dataset of moderate size, *M* can be adjusted to a higher value to mitigate the lack of image data for resampling.

### 4.11. Data-Processing Workflow of AlphaCryo4D

The following summarizes the complete procedure of AlphaCryo4D, with the detailed Algorithm 1 rationale explained in the previous subsections.
**Algorithm 1** AlphaCryo4D **Input**: Single-particle cryo-EM dataset after initial particle rejection of apparent false particles.  **Output**: Pseudo-energy landscape, MEP, 3D class assignment of each particle.  **Step 1**. Resample many 3D volumes through particle shuffling, consensus alignment and Bayesian clustering.Split the particle dataset randomly to many sub-datasets, if necessary, in case of a large dataset (e.g., >150,000 particle images), for batch processing of particle shuffling and volume resampling. Otherwise, skip this step if the dataset is small enough (e.g., <150,000 particle images).For each sub-dataset, conduct a consensus alignment to generate initial parameters of Euler angles and translations in RELION v2.1/v3.0 (MRC, Cambridge, UK) or ROME v1.1.2 (Peking University, Beijing, China) with one or more consensus models.Divide each sub-dataset into *M* + 1 groups, shuffle the sub-dataset *M* + 1 times and each time take a different group out of the shuffled sub-dataset, giving rise to *M* + 1 shuffled sub-datasets all with different collections of particles.Conduct 3D Bayesian classification on all the *M* + 1 shuffled sub-datasets to generate hundreds of 3D volumes, making each particle contribute to *M* different volumes.Execute steps (2) to (4) using the same initial model (low-pass filtered at 60-Å) for all sub-datasets.  
**Step 2**. Extract 3D feature maps of all volume data with the 3D deep residual autoencoder.Align all 3D volumes and adjust them to share a common frame of reference.Initialize the hyper-parameters of the 3D autoencoder (Table 1).Train the neural network with the 3D volume data to minimize the mean square error between the decoding layer and the input by the Adam algorithm of an initial learning rate of 0.01.Extract the 3D feature maps of all volumes from the encoding layer.  **Step 3**. Embed the volume data to two-dimensional manifolds through the t-SNE algorithm, compute the pseudo-energy landscape and find the MEP.Calculate the pairwise similarities between volumes using their feature-map-expanded volume vectors, and randomly initialize the low-dimensional points.Minimize the Kullback–Leibler divergence by the Momentum algorithm to generate 2D manifold embeddings with t-SNE.Compute the pseudo-energy landscape from the manifold using the Boltzmann relation.Initialize searching of the MEP with a straight line between given starting and ending points.Find the optimal MEP solution using the string method.  **Step 4**. Classify all particles through the energy-based particle-voting algorithm.Sample the clustering centers along the MEP and calculate the recommended clustering radius.Define the local energy minima as the 3D clustering centers and their corresponding cluster boundary for particle voting.For each particle, cast a labeled vote for a 3D class when a volume containing one of the *M* particle copies is located within the voting boundary.Count the number of votes of each particle with respect to each 3D class and assign the particle to the 3D class that receives more than *M*/2 votes from this particle.Refine each 3D density map separately to high resolution in RELION v2.1/v3.0 (MRC, Cambridge, UK) or cryoSPARC v2.9+ (Structura Biotechnology Inc., Toronto, ON, Canada) using particles classified into the same 3D classes.

### 4.12. Software Implementation

This section provides a brief account of the implementation of the AlphaCryo4D prototype system. The source code is freely available at http://github.com/alphacryo4d/alphacryo4d/ (accessed on 2 August 2022). The main programs were implemented in the Python language. Some auxiliary scripts were written in the Shell language due to its convenience. The implementation of AlphaCryo4D includes four modules, named *Resample*, *DeepFeature*, *ManifoldLandscape* and *ParticleVoting*. Table 2 summarizes all major scripts and key arguments in each module.

The programs of Bayesian resampling are provided in the folder of *Resample*. The kernel programs in Bayesian resampling include *randsf.py*, *resample.py* and *bigdata.py* (Table 2). In algorithm testing, RELION v2.1 or v3.0 (MRC, Cambridge, UK) is used in the consensus refinement and initial class assignment [3]. To speed up numerical operations, the package NumPy is called to perform mathematical calculations in the manner of vectorization. In addition, files in the *mrc* format are processed by the library *mrcfile*. If necessary, memory-mapped files are written into hard disks to break the memory limit at the cost of more running time when processing big data.

The programs of feature extraction by 3D deep residual autoencoder are provided in the folder of *DeepFeature*. The core programs to construct the 3D deep residual autoencoder and extract features of dynamics are *run_resnet.py* and *run_predict.py* (Table 2). The 3D deep residual neural network is built, trained and predicted by *Keras* with the backend of TensorFlow v1.15.4 (Alphabet Inc., Mountain View, CA, USA) [71]. The package TensorFlow can support the parallel computing of GPU to accelerate the computation, where the running environment with Compute Unified Device Architecture (CUDA) is required. Once the training of the 3D residual autoencoder is completed, the best and final network model can be saved to files of the *hdf5* format for further analysis.

The folder of *ManifoldLandscape* includes the programs *tsne_rd.py* and *string_method.py* to map the manifold embedding of 3D volumes for energy landscape estimation (Table 2). For t-SNE running in the step of manifold mapping, the package *scikit-learn* is imported to call the API of manifold learning. Besides, both the low-dimensional embedding and the energy landscape are plotted by the package *matplotlib*, which can export figures of *png* or *pdf* format. The optional calculation of the string method can generate a *npy* file of clustering centers along the MEP with the user-defined parameters.

The code in the folder of *ParticleVoting* is utilized for particle assignment in the final 3D classification using either energy-based particle voting (Figure 1D) or distance-based classification (Figure 1E). The core programs include *clustering.py* and *post_and_f.sh* for energy-based particle voting, *post_or_parallel.sh* and *dedup.sh* for distance-based classification (Table 2). Different from particle voting, the strategy of distance-based classification can retain more particle images in final 3D classes by assigning each particle to its nearest energy well without any particles being voted out. Most operations of particle assignment to 3D classes are performed by reading and writing the *star* files of particle images in parallel.

In addition, the computation of AlphaCryo4D generates multiple intermediate files and resulting output files in each module. Table 3 summarizes the Input/Output (I/O) convention of each module. The inputs and outputs of four modules are introduced with their data file formats to meet the demand for specific analysis. For compatibility with other operations in Python, most intermediate files are saved in the format of *npy*. AlphaCryo4D also exports the *star* files of particle images of each conformational state to be readily processed by RELION [3].

To configure the runtime environment for AlphaCryo4D, Python 3.7 (Python Software Foundation, Wilmington, DE, USA) is needed with the available computing environment and dependencies. The necessary dependent packages installed by conda and pip can be configured by the files *EnvConda.txt* and *EnvPip.txt* provided in the source code, respectively. Installation of RELION v2.1 or v3.0 and EMAN2 v2.91 (Baylor College of Medicine, Houston, TX, USA) is required to run through certain steps before and after AlphaCryo4D classification (Table 3) [3,72]. 

As an example, given the properly configured environment, the Python program, such as *run_resnet.py* in the folder *DeepFeature*, can be applied using the sample command like:

*python DeepFeature/run_resnet.py --batchsize 8 --validationsize 200 --regularization 0.001 --data data_dl.npy --gpu 0,1,2,3*

### 4.13. Blind Assessments with Simulated Datasets

Three simulated large datasets with the SNRs of 0.05, 0.01 and 0.005, each including 2 million particle images, were employed to benchmark AlphaCryo4D and to compare its performance with alternative methods. For each synthetic dataset, the particles were computationally simulated by projecting the 20 3D density maps calculated from 20 hypothetical atomic models emulating continuous inter-domain rotation of the NLRP3 inflammasome protein. The 20 atomic models were interpolated between the inactive NLRP3 structure and its hypothetical active state, which was generated through homology modeling using the activated NLRC4 structure (Figure 2D) [56]. The 20 atomic models represent sequential intermediate conformations during a continuous rotation in its NATCH domain against its LRR domain over an angular range of 90°. The NATCH domain in each conformation is thus rotated 4.5° over its immediate predecessor in the conformational continuum sequence; 100,000 simulated single-particle images per conformational state were generated with random defocus values in the range of −0.5 to −3.0 μm, resulting in 2 million single-particles for each dataset of a given SNR. The defocus values were not blind to all comparative tests, due to difficulty in determining accurate defocus values from single particle images of very low SNRs. The pixel size of the simulated image was set to the same as the pixel size (0.84 Å) of the real experimental dataset of the NLRP3-NEK7 complex [56]. To emulate realistic circumstances in cryo-EM imaging, Gaussian noises, random Euler angles covering half a sphere and random in-plane translational shifts from −5.0 to 5.0 pixels were then applied to every particle image.

Each simulated heterogeneous NLRP3 dataset was analyzed separately by AlphaCryo4D and used to characterize the performance and robustness of AlphaCryo4D against the variation of SNRs. In the step of particle shuffling and volume resampling, 2,000,000 particles in the dataset of any given SNR were divided randomly into 10 sub-datasets for batch processing. The orientation of each particle was determined in the initial 3D consensus alignment in RELION, which did not change in the subsequent 3D classification. In this step, the maximum number of iterations of the 3D alignment was set up as 30, with the initial reference low-pass filtered to 60 Å. Three-fold particle shuffling (indicated as × 3 below) was conducted on each sub-dataset for volume resampling. The first round of maximum-likelihood 3D classification divided the input shuffled particle sub-dataset into five classes, each of these classes was then further classified into eight classes. This procedure was separately executed on all shuffled particle sub-datasets. The particle shuffling and volume resampling generated 1372, 1489, and 1587 volumes by the datasets with SNRs of 0.05, 0.01 and 0.005, respectively. These volume data were used as inputs for deep residual autoencoder to compute low-dimensional manifolds and pseudo-energy landscapes (Figure 1D and Figure 2A–D and Supplementary Figures S1A,B). After searching the MEP on the pseudo-energy landscapes by the string method, 20 cluster centers along the MEP were defined by the local energy minima along the MEP by the approximately equal geodesic distance between adjacent minima, which represent potentially different conformations of the macromolecule (Supplementary Figure S1A–C). The particle-voting algorithm was applied in every cluster to determine the final particle sets for all 3D classes. For validation of the methodology and investigation of 3D classification improvement, we labeled each resampled 3D volume with the conformational state that held the maximum proportion of particles in the class and computed its 3D classification precision as the ratio of the particle number belonging to the labeled class versus the total particle number in the volume (Figure 2E and Figure 3).

### 4.14. Comparisons with Alternative Methods

Using 3D classification precision as a benchmark indicator, the performance of AlphaCryo4D preprocessed by standardization or 5 Å low-pass filtering was compared with several other methods: (1) the conventional maximum-likelihood-based 3D (ML3D) classification in RELION v3.0 (MRC, Cambridge, UK) [3,4,6] with and without a hierarchical strategy, (2) the conventional heterogeneous reconstruction in cryoSPARC v2.9+ (Structura Biotechnology Inc., Toronto, ON, Canada) [2], (3) the 3DVA algorithm with two and three principal components (PCs) in cryoSPARC v2.9+ [11] and (4) the deep generative model-based cryoDRGN v0.3.1 (Massachusetts Institute of Technology, Cambridge, MA, USA) [12]. A total of 24 comparative tests by these alternative methods have been conducted blindly using the three simulated datasets, which include 6 million images in total. In all our comparative tests on AlphaCryo4D and the alternative methods, the ground-truth information of particle orientations, in-plane translations and conformational identities were completely removed from the methods being tested and were not used for any steps of data processing or training. The ground-truth conformational identities of particles were only used as validation references to compute the 3D classification precisions of the blind testing results.

For the tests using ML3D in RELION, we classified all particles directly into 20 classes and hierarchically into 4 × 5 classes, which first divided the dataset into four classes, with each class further classified into five sub-classes (Figure 3). To generate the initial model for RELION, 20 ground truth density maps were averaged and low-pass filtered to 60 Å prior to ML3D; 30 iterations of ML3D classifications were then performed. For testing the conventional discrete ab initio heterogeneous reconstructions in cryoSPARC, each synthetic dataset was directly classified into 20 classes without providing any low-resolution reference model. For comparison with the 3DVA algorithm in cryoSPARC, we first conducted the blind consensus alignment of the entire dataset to find the orientation of each particle. Then the alignment and the mask generated from the consensus reconstruction were used as inputs into the 3DVA calculation, with the number of orthogonal principal modes being set to 2 or 3 in 3D classification. The 3D variability display module in the cluster mode was used to analyze the results of 3D classification. For blind tests with cryoDRGN, a default 8D latent variable model was trained for 25 epochs. The encoder and decoder architectures were 256 × 3, as recommended by the cryoDRGN developers [12]. The particle alignment parameters prior to cryoDRGN training were obtained by the same blind consensus refinement in RELION used for other parallel tests. The metadata of 3D classification precisions as well as the 3D density maps from all the algorithms applied to the three simulated datasets were collected to conduct the statistical analysis (Figure 2, Figure 3, Figure 4 and Figure 5).

### 4.15. Computational Costs

Although the computational cost of AlphaCryo4D is potentially higher than the conventional approach, it does not appear to increase drastically and likely falls in an affordable range, while reducing the average cost of computation per conformational state (Table 4). In a nutshell, we can have a brief comparison of the computational efficiency of the simulated 2 million image dataset with an SNR of 0.01. In the step of 3D data resampling, we split the dataset into 20 subsets, which contained 100,000 particles each. The 3D consensus alignment of all 2,000,000 particles costs about 75 h using eight Tesla V100 GPUs interconnected with the high-speed NVLink data bridge in an NVIDIA DGX-1 supercomputing system. Within each subset, the 3D Bayesian classification for one leave-one-group-out dataset cost about 2.5 h using 320 CPU cores (Intel Xeon Gold 6142, 2.6 GHz, 16-core chip), so the total time spent in one subset was about 10 h using 320 CPU cores in an Intel processor-based HPC cluster. In addition, it spent about 3 h extracting features via a deep neural network using eight Tesla V100 GPUs of the NVIDIA DGX-1 system. In comparison to about 160 h cost in traditional classification methods, this approach costs about 213 h using eight Tesla V100 GPUs and 320 CPU cores. As shown in Table 4, the majority of the computational cost was spent on the steps of consensus alignment and high-resolution refinement, but not on the steps of machine learning which adds overall only ~20% of the complete data processing time. We have recently optimized the ML3D code-executing efficiency for CPU-based clusters in a new version of ROME v1.1.2 [5] to speed up the consensus alignment by three- to six-fold, which will be described in detail elsewhere. Taken together, the computational cost of AlphaCryo4D is well justified, considering the output of more high-resolution conformers yielded by the procedures. In fact, in a recent study combining AlphaCryo4D with time-resolved cryo-EM [63], the actual data processing time was comparable to a similarly large dataset [25], while obtaining twice more high-resolution conformers.

### 4.16. Applications to the Experimental Cryo-EM Dataset of the Human 26S Proteasome

The substrate-engaged human 26S proteasome dataset (EMPIAR-10669) [25] includes 3,254,352 RP-CP particles (combined with particle images from both the doubly capped and singly capped proteasomes) in total, with the super-resolution counting mode pixel size of 0.685 Å and the undecimated box size of 600 × 600 pixels. The substrates of the 26S proteasome first appear in the state E_A2_. In this focused study, 147,108 particles of E_A2_ were utilized for computing the specific pseudo-energy landscape with 40 volumes. The alignment of these particles was firstly refined with a 19S mask. *M* = 3 particle shuffling was then conducted to resample the 3D volumes with a soft mask of 19S. By clustering on the pseudo-energy landscape and particle voting, two classes containing 99,043 and 47,389 particles were generated for high-resolution refinement using RELION v3.0, which were designated state E_A2.1_ and E_A2.0_, respectively. The density map of state E_A2.0_ exhibits a conformation identical to state E_A2_ in the previous report [25].

### 4.17. Applications to the Experimental Cryo-EM Dataset of the Pf80S Ribosome

The *Pf*80S ribosome dataset (EMPIAR-10028) contains 105,247 particle images. First, the alignment of all particles was refined with a global mask in RELION. To resample enough 3D volumes for the pseudo-energy landscape, we set *M* = 7 in the particle shuffling step, resulting in 79 volumes in total with a global mask and 15 Å resolution limit in the expectation step. Moreover, the regularization coefficient was set to 0.001 when training the 3D residual network with this small dataset. All volumes were then low-pass filtered at 5 Å prior to manifold embedding. Five clusters were obtained from the pseudo-energy landscape, which had 66,035, 21,482, 9922, 6424, and 657 particle images, respectively, after voting. All these clusters were refined independently using RELION v3.0.

### 4.18. Applications to the Experimental Cryo-EM Dataset of the Yeast Pre-Catalytic Spliceosome

The pre-catalytic spliceosome dataset (EMPIAR-10180) with the particle number of 327,490 shows high conformational dynamics. The consensus alignment of these particles in the original dataset was used in the first step. *M* = 7 particle shuffling was utilized to resample 160 volumes with a soft global mask and 15 Å resolution limit in the expectation step. The regularization coefficient of 0.001 was set to train the 3D Autoencoder with this dataset. All volumes were then low-pass filtered at 5 Å prior to manifold embedding. Based on the pseudo-energy landscape, we obtained 14 classes with the particle number of 15,749, 17,703, 27,522, 13,820, 20,841, 14,989, 16,377, 22,047, 25,049, 21,327, 18,949, 6157, 9790 and 36,878 after voting. Each class was refined independently using RELION v3.0.

### 4.19. Applications to the Experimental Cryo-EM Dataset of the Bacterial 50S Ribosomal Intermediates

The 131,899 particle images of the 50S ribosomal large subunit (EMPIAR-10076) are highly heterogeneous. We refined the alignment of all particles with a global mask of the 50S using RELION v3.0. In the first step, 119 resampled volumes were utilized to calculate the pseudo-energy landscape. *M* = 7 particle shuffling was conducted to generate these volumes without any mask. The regularization coefficient of 0.001 was set when training the deep neural network. All volumes were then low-pass filtered at 5 Å prior to manifold embedding. Then the first pseudo-energy landscape resulted in nine clusters with the particle number of 766 (A), 15,129 (B), 1236, 322, 670, 22,037, 25,445 (D), 33,976 (E) and 25,115 (C), respectively. For the clusters of states B–E, four zoom-in pseudo-energy landscapes were then computed to discover more sub-states with 46, 56, 66 and 72 resampled volumes, respectively. In the zoom-in pseudo-energy landscape calculation, *M* = 7 was set in particle shuffling for resampling 3D volumes with a global mask and 20 Å resolution limit in the expectation step. Then the regularization coefficient of 0.001 was used to train the deep residual network. All volumes were then low-pass filtered at 5 Å prior to manifold embedding. After particle voting, the pseudo-energy landscape of state B resulted in three sub-clusters with particle numbers of 3494, 3600 and 7803. The state C pseudo-energy landscape resulted in five sub-clusters with the particle numbers of 2444, 7442, 711, 7633 and 5557. The state D pseudo-energy landscape resulted in five sub-clusters with the particle numbers of 6231, 8785, 2123, 3718 and 4092. Moreover, the pseudo-energy landscape of state E resulted in eight sub-clusters with the particle numbers of 4783, 1588, 5891, 3620, 7352, 5265, 357 and 2241. All these clusters were refined using RELION v3.0. 

## Figures and Tables

**Table 1:** Hyperparameters of the deep residual networks in the 3D autoencoder. Optimizer: Adam. Epochs: 50. Initial learning rate: 0.01. A factor of 0.1 and patience of 3 means that the learning rate will times 0.1 (factor) if the loss function does not improve in 3 (patience) epochs.

Layer Name	Output Size (Simulated Datasets)	Output Size (Proteasome)	Output Size (*pf*80S Ribosome)	Output Size (Spliceosome)	Output Size (Bacterial Ribosomal Intermediates)	Network Structure
Conv1	200×200×200	300×300×300	360×360×360	320×320×320	320×320×320	5×5×5, 2
Conv2_x	100×100×100	150×150×150	180×180×180	160×160×160	160×160×160	3×3×3, 2, stride 2 3×3×3, 2
Conv3_x						3×3×3, 2 3×3×3, 2
Conv4_x	50×50×50	75×75×75	90×90×90	80×80×80	80×80×80	3×3×3, 1, stride 2 3×3×3, 1
Conv5_x						$\begin{array}{c} 3\times 3\times 3,\;1\\ 3\times 3\times 3,\;1 \end{array}$
Conv6_x (encoding)						$\begin{array}{c} 3\times 3\times 3,\;1\\ 3\times 3\times 3,\;1 \end{array}$
TransConv7_x	100×100×100	150×150×150	180×180×180	160×160×160	160×160×160	3×3×3, 1, stride 2 3×3×3, 1
TransConv8_x						3×3×3, 1 3×3×3, 1
TransConv9_x	200×200×200	300×300×300	360×360×360	320×320×320	320×320×320	3×3×3, 2, stride 2 3×3×3, 2
TransConv10_x						3×3×3, 2 3×3×3, 2
TransConv11						5×5×5, 1

**Table 2:** Kernel code, key arguments and their interpretations of four modules in AlphaCryo4D.

Modules	Kernel Code	Key Arguments	Interpretations	Default Values
Bayesian resampling	*randsf.py*	*--star*	*Star* file name of all particles	*particles.star*
		*--number*	Particle number of each batch	*100,000*
	*resample.py*	*--fold*	Value of *M* in particle shuffling ^1^	*3*
	*bigdata.py*	*--folder*	Folder name of 3D volumes	*./maps_aligned/*
		*--std*	Whether to standardize these 3D volumes	*True*
3D deep residual autoencoder	*run_resnet.py*	*--epoch*	Autoencoder training epochs	*50*
		*--batchsize*	Batch size of 3D volumes during training	*8*
		*--validationsize*	Validation data size of 3D volumes	*800*
		*--regularization*	L2 regularization coefficient	*0*
		*--gpu*	ID of GPU utilization	*0,1,2,3*
	*run_predict.py*	*--data*	Input 3D volumes	*data_dl.npy*
		*--model*	Network model for feature extraction	*checkpoint/check.h5*
		*--batchsize*	Batch size of 3D volumes during prediction	*Result/feature.npy*
Manifold mapping	*tsne_rd.py*	*--seed*	Random seed for reproducibility	*0*
		*--perplexity*	Perplexity of t-SNE	*30.0*
		*--niter*	Maximum iterations in t-SNE	*1000*
	*string_method.py*	*--range*	Maximum value of low-dimensional coordinates on the energy landscape	*100.0*
		*--start*	Position around the start point of MEP	*−80.0–80.0*
		*--end*	Position around the end point of MEP	*80.0 80.0*
		*--stepmax*	Maximum steps in the string method	*100,000*
		*--stepsize*	Step size of the string method	*0.1*
		*--tolerance*	Convergence condition in the string method	*10^-7^*
		*--interpolate*	Interpolation method to plot energy landscape	*linear*
		*--kcenters*	Number of clustering centers along the MEP	*20*
Particle assignment	*clustering.py*	*--centers*	The *npy* file of clustering centers	*centers_k.npy*
		*--radius*	Radius of the clustering boundary	*30.0*
	*post_and_f.sh*	*th*	Threshold of particle voting	*2*
	*post_or_parallel.sh*	*\*	Code for distance-based classification	*\*
	*dedup.sh*	*\*	Code for removal of duplicated particles in the strategy of distance-based classification	*\*

^1^ The *star* files of shuffled particles are employed for reconstruction of 3D volumes in RLEION.

**Table 3:** Dependencies and I/O of four modules in AlphaCryo4D.

Dependencies	Modules	Folder	Inputs	Outputs
Python 3.7.1 TensorFlow 1.15.4 Keras 2.3.1 scikit-learn 0.24.0 mrcfile 1.2.0 NumPy 1.18.5 Matplotlib 3.3.3 RELION v2.1 or v3.0 ROME v1.1.2 EMAN2 v2.91	Bayesian resampling	*Resample*	Particle images (*star* files and *mrc* format)	3D volumes (*star* files and *npy* format)
	3D deep residual autoencoder	*DeepFeature*	3D volumes (*npy* format)	Feature maps (*npy* format)
	Manifold mapping	*ManifoldLandscape*	3D volumes (*star* files and *npy* format)	Energy landscape (*npy* and *pdf* format)
			Feature maps (*npy* format)	Optional clustering centers along the MEP (*npy* format)
	Particle voting	*ParticleVoting*	Particle images (*star* files and *mrc* format)	3D reconstructions (*star* files and *mrc* format)
			Energy landscape (*npy* format)	

**Table 4:** Approximate computational time of AlphaCryo4D (grey boxes) on the simulated 2-million image dataset with the SNR of 0.01 and three experimental datasets.

	Particle Number	Consensus Alignment by RELION (h)	3D Volumes Resampling (h)	Autoencoder Training (h)	Manifold Learning (h)	High-Resolution Refinement by RELION (h)
Simulated dataset with the SNR of 0.01	2,000,000	75	10	3	15	110
Yeast Spliceosome (EMPIAR-10180)	327,490	\	48	4	0.1	30
*Pf*80S Ribosome (EMPIAR-10028)	105,247	80	30	2	0.1	70
Bacterial Ribosome (EMPIAR-10076)	131,899	160	38	4	0.2	105

## Data Availability

The experimental data used in this study are based on the public datasets available from the raw cryo-EM data online repository EMPIAR (https://www.ebi.ac.uk/empiar/, accessed on 2 August 2022) through the accession numbers EMPIAR-10028, EMPIAR-10076, EMPIAR-10180, and EMPIAR-10669. The simulated datasets are available upon request from the corresponding author. Source code of AlphaCryo4D is available at http://github.com/alphacryo4d/alphacryo4d/ (accessed on 2 August 2022). Source code of ROME is available at http://github.com/alphacryo4d/rome/ (accessed on 2 August 2022).

## Supplementary Materials

The supporting information can be downloaded at: https://www.mdpi.com/article/10.3390/ijms23168872/s1.
