# Supplementary material for: Visualizing Conformational Space of Functional Biomolecular Complexes by Deep Manifold Learning
Source: Int J Mol Sci. 2022 Aug 9;23(16):8872. doi: 10.3390/ijms23168872 (PMC9408802; doi:10.3390/ijms23168872)
Supplement: Supplementary file 1 [file ijms-23-08872-s001.zip › Supp/Supplementary_Information.pdf]

Supplementary Materials

Visualizing Conformational Space of Functional Biomolecular Complexes by Deep Manifold Learning

Zhaolong Wu [1,2,3], Enbo Chen [1,2], Shuwen Zhang [1,2], Yinping Ma [4] and Youdong Mao [1,2,3,5,*]

[1] State Key Laboratory for Artificial Microstructure and Mesoscopic Physics, School of Physics, Peking University, Beijing 100871, China
[2] Peking-Tsinghua Joint Center for Life Sciences, Peking University, Beijing 100871, China
[3] Center for Quantitative Biology, Peking University, Beijing 100871, China
[4] Computing Center, Peking University, Beijing 100871, China
[5] National Biomedical Imaging Center, Peking University, Beijing 100871, China
* Correspondence: ymao@pku.edu.cn

The Supplementary Materials include 7 Supplementary Figures (S1-S7) and 4 Videos (S1-S4).

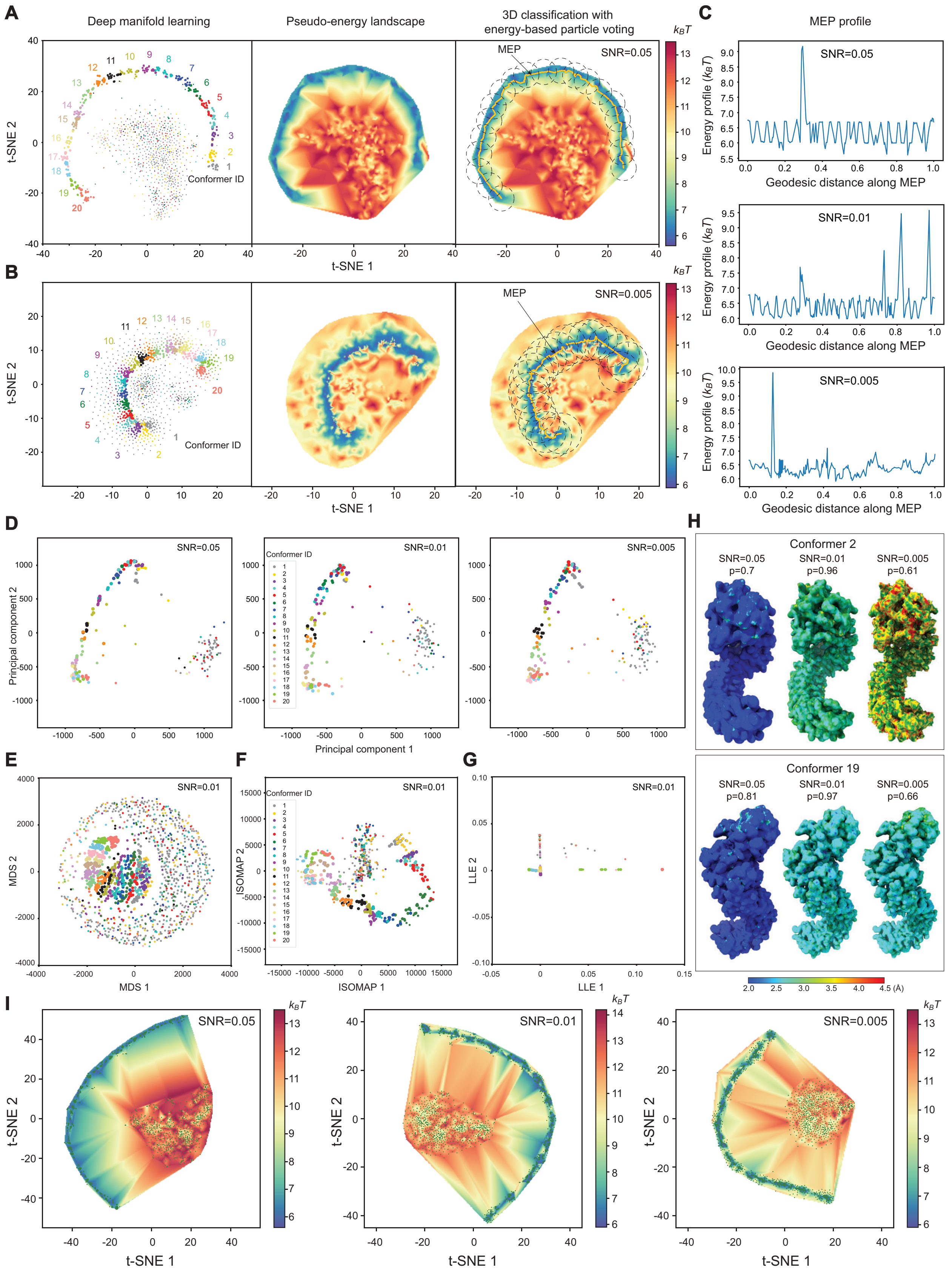

A
Deep manifold learning
Pseudo-energy landscape
3D classification with energy-based particle voting
k_BT
MEP
SNR=0.05
Conformer ID
t-SNE 1
t-SNE 2
B
MEP
SNR=0.005
Conformer ID
C
MEP profile
SNR=0.05
SNR=0.01
SNR=0.005
Energy profile (k_BT)
Geodesic distance along MEP
D
SNR=0.05
SNR=0.01
SNR=0.005
Conformer ID
Principal component 1
Principal component 2
E
SNR=0.01
MDS 1
MDS 2
F
SNR=0.01
Conformer ID
ISOMAP 1
ISOMAP 2
G
SNR=0.01
LLE 1
LLE 2
H
Conformer 2
SNR=0.05 p=0.7
SNR=0.01 p=0.96
SNR=0.005 p=0.61
Conformer 19
SNR=0.05 p=0.81
SNR=0.01 p=0.97
SNR=0.005 p=0.66
2.0 2.5 3.0 3.5 4.0 4.5 (Å)
I
SNR=0.05
SNR=0.01
SNR=0.005
k_BT
t-SNE 1
t-SNE 2

Supplementary Figure S1. Blind assessments of AlphaCryo4D and its comparison with the 3D PCA method using the simulated heterogeneous NLRP3 datasets of different SNRs. (**A**) and (**B**) Reconstruction of the pseudo-energy landscape of the simulated NLRP3 datasets at SNRs of 0.05 (**A**) and 0.005 (**B**) by the t-SNE algorithm using the resampled volumes and their corresponding feature maps. Colors in the left panels indicate the ground truth of 3D volume data points. (**C**) Free energy profiles along the MEP calculated by the string method in the 2D pseudo-energy landscapes of the simulated NLRP3 datasets at SNRs of 0.05 (top), 0.01 (middle) and 0.005 (bottom). (**D**) Linear dimensionality reduction of resampled 3D volumes from the simulated datasets at three distinct SNRs by the 3D PCA method. (**E**-**G**) Dimensionality reduction of resampled 3D volumes from the simulated dataset at SNR of 0.01 by the multidimensional scaling (MDS) (panel **E**), isometric mapping (Isomap) (panel **F**) and locally linear embedding (LLE) algorithms (panel **G**). Colors of data points indicate the ground truth of their corresponding 3D volumes. (**H**) Comparison of local resolution assessment of AlphaCryo4D-classified NLRP3 reconstructions of conformers 2 (upper inset) and 19 (lower inset) from the simulated datasets of three distinct SNRs. The local resolutions were computed by ResMap. Conformers 2 and 19 have been missed 10 and 8 times in 18 control tests using several other methods (see Figure 3), which are the most and second-most frequently missed conformers, respectively. The 3D classification precision (P) is labelled above each density map. The color bar of local resolution is shown in the lower insert. **i,** Pseudo-energy landscape calculated by 3D volumes with preprocessing of 5 Å low-pass filtering instead of standardization. The gap between the majority and minority data points was widened at different SNRs.

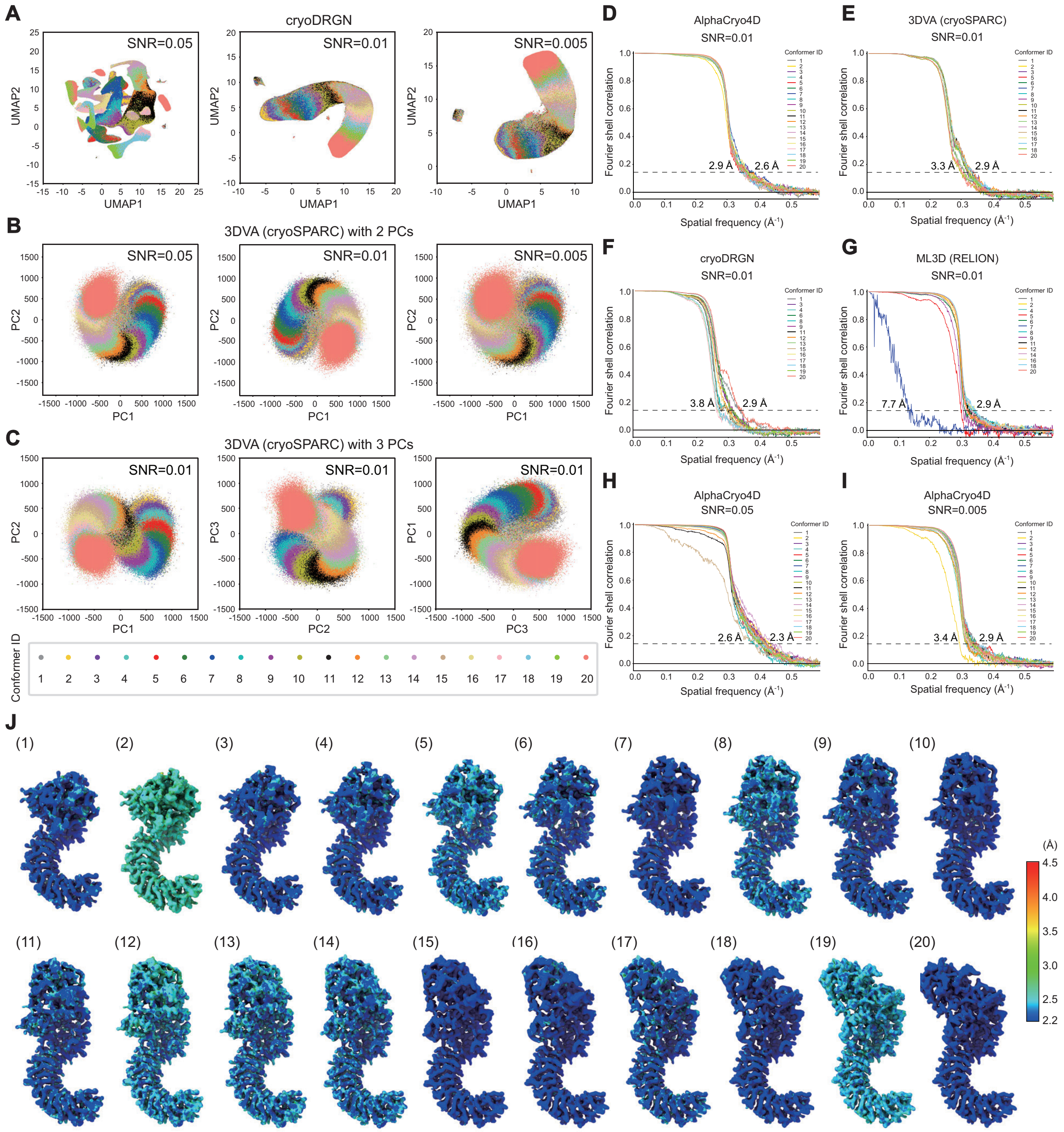

Supplementary Figure S2. Performance comparison of AlphaCryo4D with alternative methods using the simulated heterogeneous NLRP3 datasets of different SNRs. (**A-C**) Visualization of 3D classification by cryoDRGN in autoencoder-learned feature space (**A**), and by 3DVA with 2 PCs (**B**) and 3 PCs (**C**). (**D-G**) The gold-standard FSC plots of the 20 maps resulting from 3D classification by AlphaCryo4D (**D**), of the 18 maps resulting from the 3D classification by 3DVA in cryoSPARC (**E**), of the 15 maps resulting from cryoDRGN (**F**) and of the 14 maps resulting from the maximum-likelihood 3D classification in RELION (**G**) on the simulated data of 0.01 SNR. (**P**) and (**Q**) Additional gold-standard FSC plots of the refined density maps resulting from AlphaCryo4D on the simulated datasets of SNRs of 0.05 and 0.005. (**J**) The local resolution maps of the 20 refined conformers from AlpahCryo4D corresponding to Figure 2E.

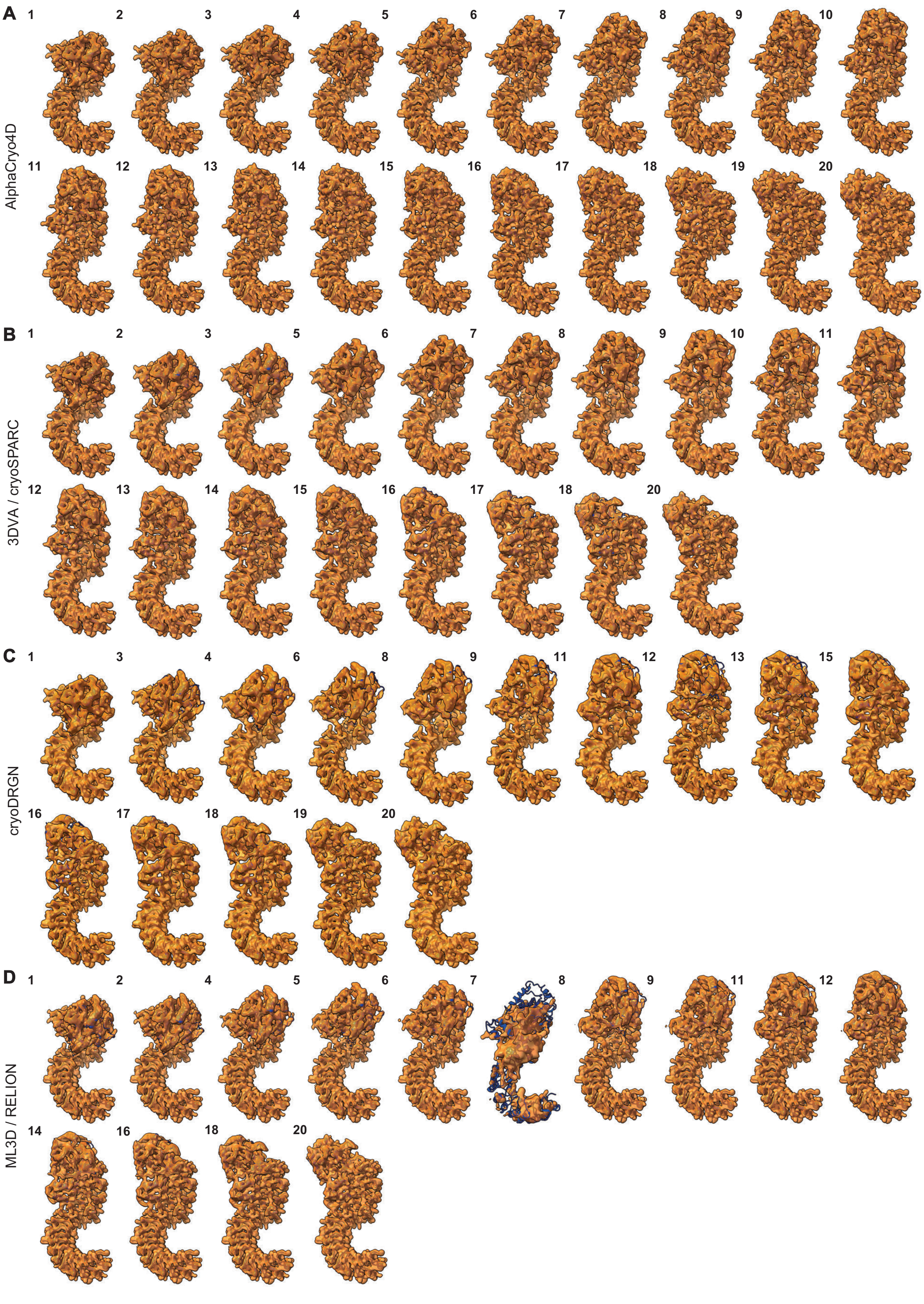

A
AlphaCryo4D
1 2 3 4 5 6 7 8 9 10
11 12 13 14 15 16 17 18 19 20
B
3DVA / cryoSPARC
1 2 3 5 6 7 8 9 10 11
12 13 14 15 16 17 18 20
C
cryoDRGN
1 3 4 6 8 9 11 12 13 15
16 17 18 19 20
D
ML3D / RELION
1 2 4 5 6 7 8 9 11 12
14 16 18 20

Supplementary Figure S3. Map assessments of AlphaCryo4D in reconstructions of conformational continuum in comparison with conventional methods on the simulated data of 0.01 SNR. (**A**) The 20 maps of distinct NLRP3 conformers resulting from the 3D classification by AlphaCryo4D. (**B**) The 18 maps of NLRP3 resulting from 3DVA in cryoSPARC. (**C**) The 15 maps of NLRP3 resulting from the 3D classification by cryoDRGN. (**D**) The 14 maps of NLRP3 resulting from the 3D classification by ML3D in RELION. All maps are shown in transparent surface representations superimposed with their corresponding atomic models of the ground truth in cartoon representations, which are fitted to the maps as rigid bodies without further atomic modelling. The conformer ID numbers are marked on the upper left of each map panel. The results shown in panels (**A**), (**B**), (**C**) and (**D**) correspond to the FSC results shown in panels (**E**), (**F**), (**G**) and (**H**) of Figure 2, respectively.

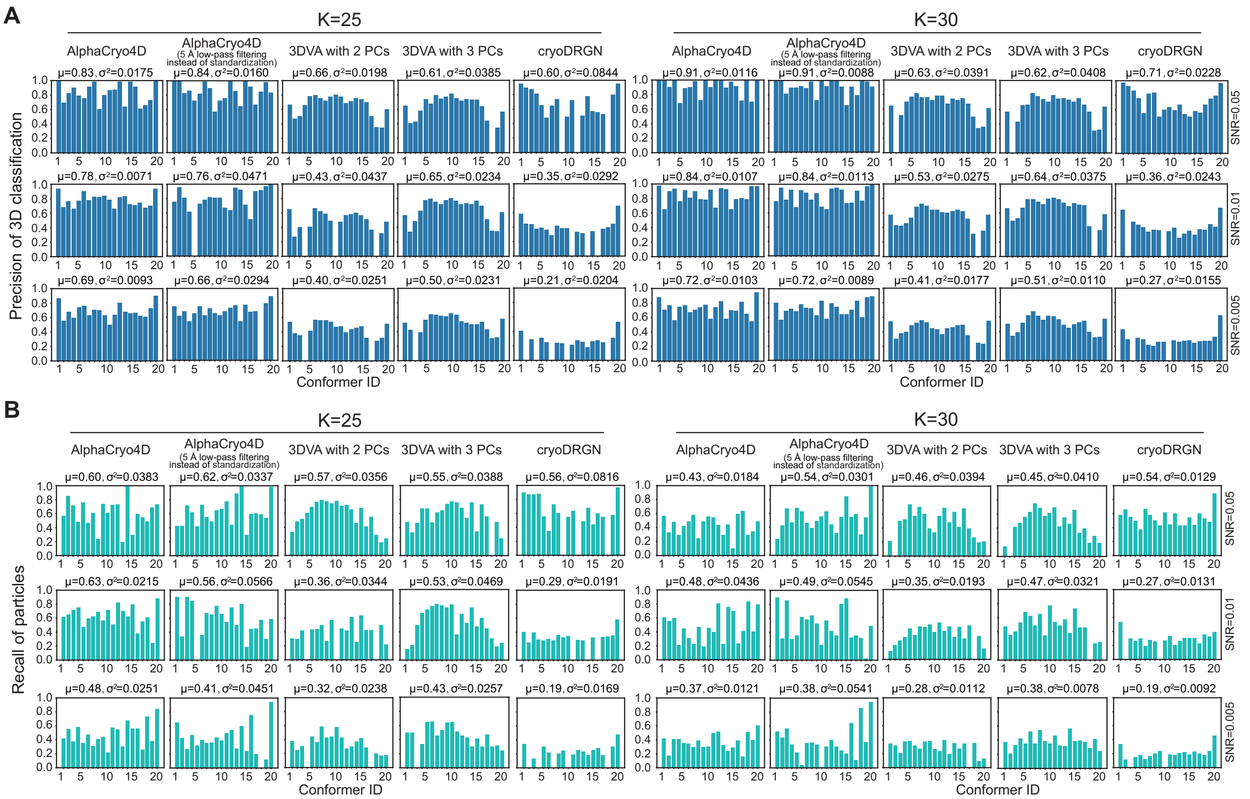

Supplementary Figure S4. Performance evaluation of 3D classification with alternative parameters and comparison of the recall of 3D classification using the simulated datasets of three distinct SNRs. (**A**) 3D classification precision of AlphaCryo4D preprocessed by standardization or 5 Å low-pass filtering, 3DVA using two PCs, 3DVA using three PCs and cryoDRGN when the class number K was set to 25 or 30, which are more than the class number 20 of ground truths. (**B**) 3D classification recall of AlphaCryo4D preprocessed by standardization or 5 Å low-pass filtering, 3DVA using two PCs, 3DVA using three PCs and cryoDRGN when the class number was set to 25 or 30 (more than the class number 20 of ground truths).

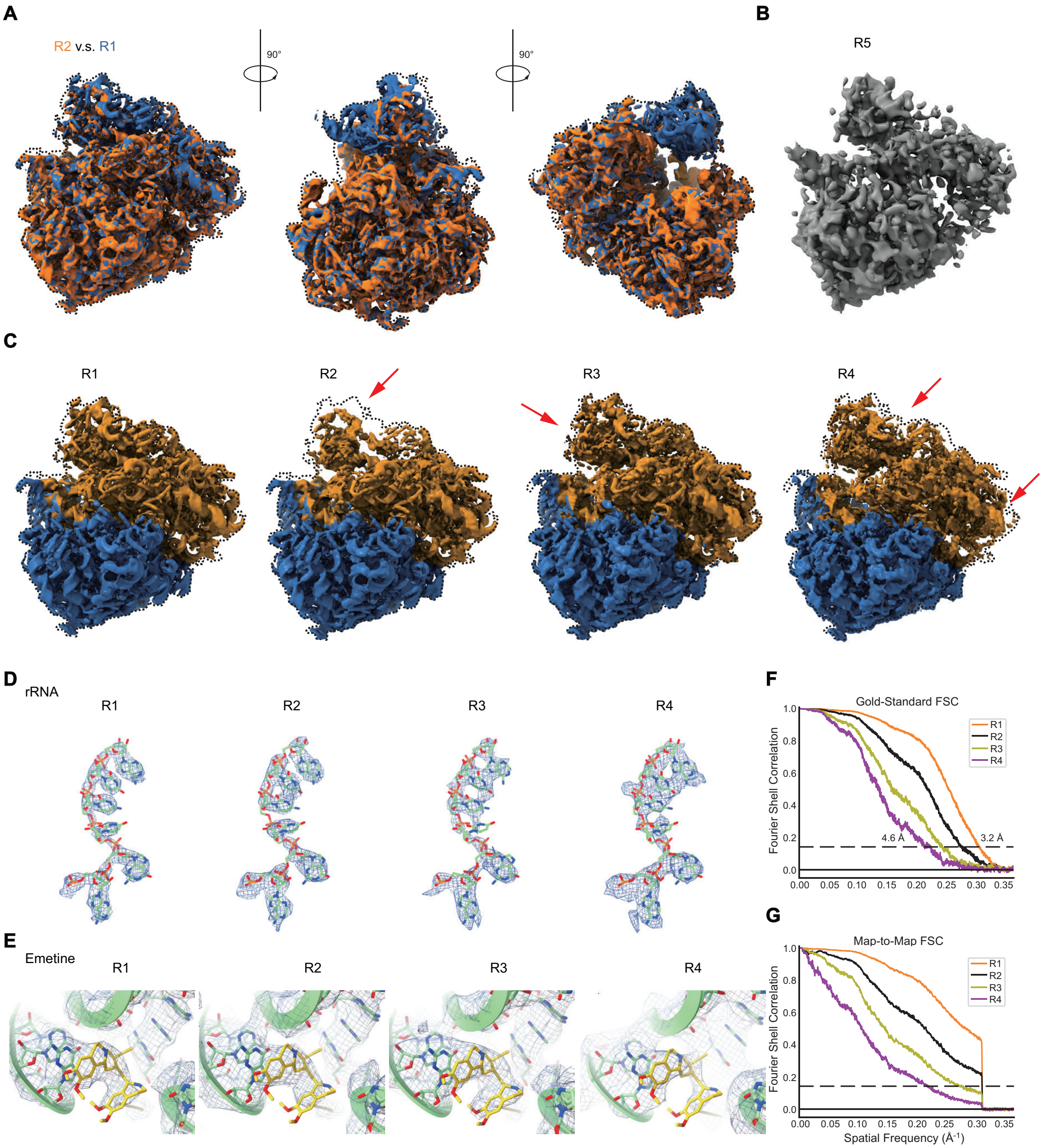

Supplementary Figure S5. Heterogeneous analysis of the *Pf*80S ribosome reconstructed by AlphaCryo4D. (**A**) Cryo-EM density map of the 40S head missing state R2 superimposed with the major state R1 from three different viewing angles. (**B**) Cryo-EM density map of cluster R5 on the pseudo-energy landscape (Figure 7A). The particle number of this cluster limited the resolution of its final reconstructed density map. (**C**) Comparison of the density maps of clusters R1-R4. The red arrows point to the conformational difference of states R2-R4 relative to the state R1. (**D**) Comparison of local cryo-EM densities of an rRNA strand superimposed with its corresponding atomic model in four conformational states. (**E**) Comparison of local cryo-EM densities of the small-molecule drug emetine bound to the ribosome, superimposed with its corresponding atomic model, in four conformational states. (**F**) Gold-standard FSC of the R1-R4 states reconstructed by AlphaCryo4D. (**G**) Map-to-map FSC of the R1-R4 states with the published cryo-EM structure of the *Pf*80S ribosome (EMD-2660). The cliff of the FSC curve was caused by the low-pass filtering of the previously released cryo-EM maps.

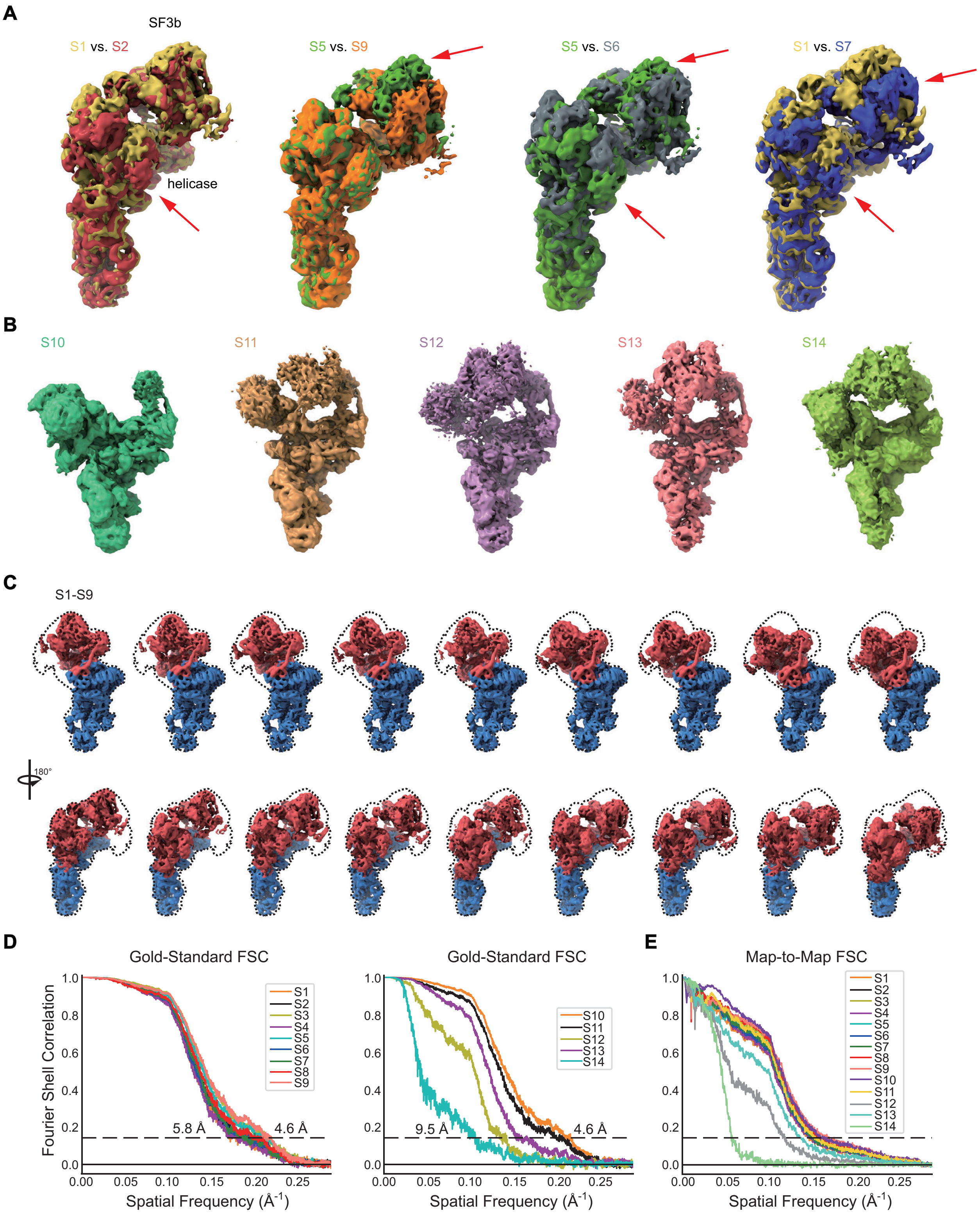

Supplementary Figure S6. Multiple motion patterns of pre-catalytic spliceosome. (**A**) Different local motion modes of the SF3b and helicase subcomplexes. The red arrows pointed the regions where the conformational changes are observed by comparing the cryo-EM densities of different states. These four comparisons exhibited the local

movement of helicase (S1 vs. S2), the local movement of SF3b (S5 vs. S9), the relative motions of the SF3b and helicase subcomplexes in the opposite direction (S5 vs. S6) and the concerted motions of the SF3b and helicase subcomplexes in the same direction (S1 vs. S7), which showed that the continuous motion the SF3b or helicase subcomplexes is independent. (**B**) Cryo-EM density maps of clusters S10-S14. Discrete heterogeneity is observed in these states. (**C**) Cryo-EM density maps of clusters S1-S9 from other two viewing angles. The dynamic region of the density map is colored red and the rigid region is colored blue. Continuous conformational motion was observed among these states. (**D**) Gold-standard FSC of the S1-S14 states classified by AlphaCryo4D. (**E**) Map-to-map FSC of the S1-S14 states over the published B4 cryo-EM density map of the pre-catalytic spliceosome (EMD-3685).

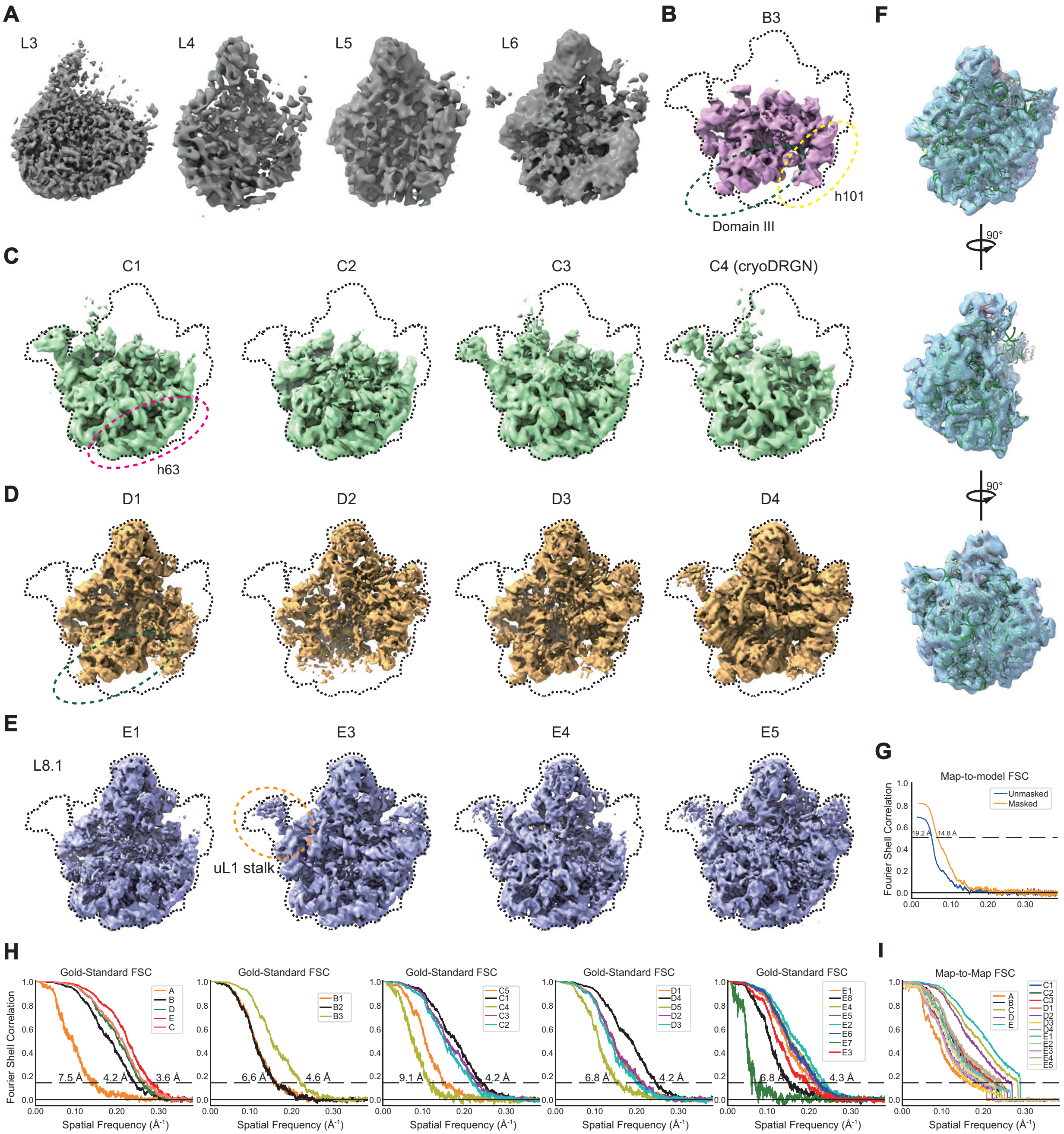

Supplementary Figure S7. Reconstructions of bacterial ribosomal assembly intermediates by AlphaCryo4D. (**A**) Cryo-EM density maps of clusters L3-L6. The classes contained particles of likely poor quality. **b,** Reconstruction of the cluster B3 from the state B pseudo-energy landscape (Figure 9D). The cluster B3 revealed the main conformation identical to state B. (**C**) Reconstructions of the state C1, C2, C3 and C4 from the state C pseudo-energy landscape (Figure 9F). The state C4 contained the density of rRNA helix 68, which was previously found by cryoDRGN. (**D**) Reconstructions of the state D1, D2, D3 and D4 from the state D pseudo-energy landscape (Figure 9H). (**E**) Reconstructions of the state E1, E3, E4 and E5 from the state E pseudo-energy landscape (Figure 10J). (**F**) The cryo-EM map of the state E7 in transparent surface representation superimposed with corresponding atomic model in cartoon representation from three different viewing angles. (**G**) Model-to-map FSC plots of the state E7 reconstruction with and without applying a global mask. (**H**) Gold-standard FSC of the 5 major states (A, B, C, D and E) and 21 minor states (B1-B3, C1-C5, D1-D5 and E1-E8) reconstructed by AlphaCryo4D. (**I**) Map-to-map FSC of

the existing states over the published cryo-EM structures of the states A (EMD-8434), B (EMD-8440), C (EMD-8441), D (EMD-8445), E (EMD-8450), C1 (EMD-8442), C2 (EMD-8443), C3 (EMD-8444), D1 (EMD-8446), D2 (EMD-8447), D3 (EMD-8448), D4 (EMD-8449), E1 (EMD-8451), E2 (EMD-8452), E3 (EMD-8453), E4 (EMD-8455) and E5 (EMD-8456) of the bacterial ribosomal assembly intermediates.

Supplementary Video S1. Discovery of a new intermediate state of the 26S proteasome by AlphaCryo4D.

Supplementary Video S2. Visualizing conformational dynamics of the *Pf*80S ribosome bound to anti-protozoan drug by AlphaCryo4D.

Supplementary Video S3. Choreographing continuous inter-subunit motions of the pre-catalytic spliceosome by AlphaCryo4D

Supplementary Video S4. Exploring hidden conformational space of the bacterial ribosomal assembly intermediates by AlphaCryo4D.
